# *OsPGIP1*-Mediated Resistance to Bacterial Leaf Streak in Rice is Beyond Responsive to the Polygalacturonase of *Xanthomonas oryzae* pv. *oryzicola*

**DOI:** 10.1186/s12284-019-0352-4

**Published:** 2019-12-12

**Authors:** Tao Wu, Chune Peng, Beibei Li, Wei Wu, Lingguang Kong, Fuchuan Li, Zhaohui Chu, Fang Liu, Xinhua Ding

**Affiliations:** 1 State Key Laboratory of Crop Biology, College of Plant Protection, Shandong Agricultural University, Tai’an, 271018 Shandong China; 2 College of Life Science, Shandong Agricultural University, Tai’an, 271018 Shandong China; 3 National Glycoengineering Research Center and State Key Laboratory of Microbial Technology, Shandong University, Jinan, 250100 Shandong China; 4 College of Agronomy, Shandong Agricultural University, Tai’an, 271018 Shandong China; 5 Key Laboratory of Oil Crop Biology of the Ministry of Agriculture, Chinese Academy of Agricultural Sciences, Oil Crops Research Institute, Wuhan, 430062 China

**Keywords:** Bacterial leaf streak, Cell wall-associated genes, Defense, Jasmonic acid, Polygalacturonase-inhibiting proteins, Rice, Sheath blight

## Abstract

Polygalacturonase-inhibiting proteins (PGIPs) have been shown to recognize fungal polygalacturonases (PGs), which initiate innate immunity in various plant species. Notably, the connection between rice OsPGIPs and PGs in *Xanthomonas oryzae* pv. *oryzicola* (*Xoc*), which causes bacterial leaf streak (BLS), remains unclear. Here, we show that *OsPGIP1* was strongly induced after inoculating rice with the *Xoc* strain RS105. Furthermore, *OsPGIP1*-overexpressing (OV) and RNA interference (RNAi) rice lines increased and decreased, respectively, the resistance of rice to RS105, indicating that *OsPGIP1* contributes to BLS resistance. Subsequently, we generated the unique PG mutant RS105Δpg, the virulence of which is attenuated compared to that of RS105. Surprisingly, the lesion lengths caused by RS105Δpg were similar to those caused by RS105 in the OV lines compared with wild-type ZH11 with reduced *Xoc* susceptibility. However, the lesion lengths caused by RS105Δpg were still significantly shorter in the OV lines than in ZH11, implying that *OsPGIP1*-mediated BLS resistance could respond to other virulence factors in addition to PGs. To explore the *OsPGIP1*-mediated resistance, RNA-seq analysis were performed and showed that many plant cell wall-associated genes and several MYB transcription factor genes were specifically expressed or more highly induced in the OV lines compared to ZH11 postinoculation with RS105. Consistent with the expression of the differentially expressed genes, the OV plants accumulated a higher content of jasmonic acid (JA) than ZH11 postinoculation with RS105, suggesting that the *OsPGIP1*-mediated resistance to BLS is mainly dependent on the plant cell wall-associated immunity and the JA signaling pathway.

## Background

The battle between pathogens and plants, known as the “arms race”, is the result of millions of years of coevolution (Boller and He 2009). Similar to the skin of animals, the plant cell surface is the first layer of physical and chemical protection against invading pathogens. This first protective layer includes the plant waxy cuticles and the release of plant metabolites that act as anti-microbial compounds (Jones and Dangl 2006; Malinovsky et al. 2014). Unlike animals with circulating antibodies against pathogens, plants have developed a two-tiered innate immune systems: pattern-triggered immunity (PTI) and effector-triggered immunity (ETI) (Jones and Dangl 2006; Li et al. 2016). Beyond the cuticle layer, the plant cell wall is the second barrier that prevents the colonization of phytopathogenic organisms (Bellincampi et al. 2014). The plant cell well is a dynamic structure that is mainly composed of a framework of cellulose microfibrils that are cross-linked to each other by heteropolysaccharides to create a rigid structural framework (Lampugnani et al. 2018). To penetrate the plant cell wall and colonize their host, plant pathogens produce cell wall-degrading enzymes (CWDEs), including pectin methylesterases (PMEs), hemicellulases, cellulase and polygalacturonases (PGs) (Kalunke et al. 2015). PGs are secreted by fungi, bacteria and insects at the early stage of infection and serve as a pathogenicity factor. This hydrolytic enzyme cleaves the α-(1–4) linkages between the D-galacturonic acid residues of homogalacturonan to degrade cell wall polysaccharides and facilitate the availability of host nutrients (Kalunke et al. 2015; Bacete et al. 2018).

To prevent the degradation of the cell wall by phytopathogens and insects, plants induce the expression of CWDE inhibitors, such as polygalacturonase-inhibiting proteins (PGIPs), to block the activity of PG, delaying the hydrolysis of oligogalacturonides (Kalunke et al. 2015). PGIPs are typically plant cell wall proteins that contain ten imperfect leucine-rich repeat (LRR) motifs to form two β-sheets that interact with PGs (Di Matteo et al. 2003; Benedetti et al. 2011). In most cases, plant PGIPs show inhibitory activity against PGs in vitro, suggesting that they encode defense-related genes (Wang et al. 2015a; Kalunke et al. 2015). In addition to directly inhibiting PGs, PGIPs can form a complex with PGs, to promote the generation of oligogalacturonide (OG) fragments with low degrees of polymerization (DP) (Benedetti et al. 2015). OGs function as a damage-associated molecular pattern (DAMP) that is recognized by the receptor wall-associated kinase 1 (WAK1) to induce host immunity (Brutus et al. 2010). Studies have indicated that the application of long or trimeric OGs activates the immune response to resist necrotrophic pathogens and nematodes (Galletti et al. 2008; Rasul et al. 2012; Davidsson et al. 2017; Shah et al. 2017).

PGIPs have been shown to be regulators of resistance to different pathogens in a variety of plants. Overexpressing *PcPGIP* from pear and *BrPGIP2* from *Brassica rapa* resulted in enhanced resistance to the bacterial pathogens *Xylella fastidiosa* and *Pectobacterium carotovorum* in grapevine and Chinese cabbage, respectively (Agüero et al. 2005; Hwang et al. 2010). Numerous plant PGIPs, including PvPGIP2 from *Phaseolus vulgaris* (Sicilia et al. 2005), PGIP from tomato (Schacht et al. 2011), PGIP from bean (Borras-Hidalgo et al. 2012), GmPGIP3 from *Glycine max* (Wang et al. 2015a), VrPGIP2 from mungbean (Chotechung et al. 2016) and GhPGIP1 from cotton (Liu et al. 2017), play positive roles in the resistance to different fungi, partially by suppressing PG activity. In *Oryza sativa*, the expression of five out of seven *PGIP* genes is upregulated in response to *Rhizoctonia solani* infection, the causative agent of sheath blight (SB) of rice (Lu et al. 2012). *OsPGIP1* was identified to positively regulate resistance through the direct inhibition of PGs produced by *R. solani* (Wang et al. 2015b; Chen et al. 2016). Furthermore, the expression of *OsPGIP4* was reported to be upregulated upon bacterial pathogen infection, and overexpressing *OsPGIP4* in rice enhanced the resistance of rice to bacterial leaf streak (BLS) (Feng et al. 2016).

BLS, which is caused by *Xanthomonas oryzae* pv. *oryzicola* (*Xoc*), is one of the major bacterial diseases of rice, and it is difficult to control due to the lack of highly disease-resistant rice varieties (Niño-Liu et al. 2006). BLS is prevalent among southern and central China, southeast Asia and African, with increasing outbreak frequency and severity. Two sources of resistance have been documented for BLS: the qualitative resistance gene locus *Xo1*, which is only effective against African *Xoc* isolates, and the quantitative trait loci (QTL) *qBlsr5a* (*xa5*), which confers resistance to both BLS and bacterial blight of rice (Xie et al. 2014; Triplett et al. 2016). Research has shown that modifying the expression of defense-related (*DR*) genes alters the resistance of rice to *Xoc*. For example, the overexpression of the broad-spectrum disease resistance gene *OsMPK6*, an indole-3-acetic acid amido synthetase (*GH3–2*), a nucleotide binding and leucine-rich repeat domain (NLR) protein heteropairs *RGA4/RGA5*, *OsPGIP4*, *OsMAPK10.2*, the small heat shock protein gene *OsHSP18.0-CI* and the phytosulfokine receptor 1 (*OsPSKR1*) enhanced the resistance of rice to BLS (Shen et al. 2010; Fu et al. 2011; Feng et al. 2016; Hutin et al. 2016; Ma et al. 2017; Ju et al. 2017; Yang et al. 2019). The repression of *OsWRKY45–1*, a receptor-like cytoplasmic kinase (*NRRB*), *OsImpα1a* and *OsImpα1b* also enhanced the resistance of rice to *Xoc* (Tao et al. 2009; Guo et al. 2014; Hui et al. 2019).

We previously reported that overexpression of *OsPGIP4* enhanced the resistance of rice to BLS (Feng et al. 2016); however, the role and mechanism of action of OsPGIP1 in rice and *Xoc* interactions remains unknown. In this study, we showed that *OsPGIP1* expression was induced in response to *Xoc*. We generated *OsPGIP1*-overexpressing and *OsPGIP1*-suppressed transgenic rice and demonstrated the positive role of *OsPGIP1* in the resistance of rice to BLS. Unlike previous examples of the PGIP-PGs working model, the *OsPGIP1*-mediated resistance to BLS is induced by other pathogenicity factors in addition to the PG of *Xoc*. Moreover, our results showed that the *OsPGIP1*-mediated immune response to BLS was related to pathogen-related (*PR*) gene and cell wall-associated gene expression through RNA sequencing analysis and jasmonic acid (JA) accumulation. In general, our results demonstrate the benefits of utilizing *OsPGIP1* in breeding disease-resistant rice that will be resistant to BLS and SB caused by bacterial and fungal pathogens, respectively.

## Methods

### Plant Materials, Bacterial Strains, Plasmids and Rice Transformation

The BLS-susceptible rice variety Zhonghua 11 (ZH11, *Oryzae sativa* L. ssp. *japonica*) and moderately resistant variety Acc8558 (*O. sativa* L. ssp. *indica*), the donor of the BLS-resistance quantitative trait locus *qBlsr5a* that contains *xa5*, *OsPGIP1* and *OsPGIP4* (Chen et al. 2006; Xie et al. 2014; Feng et al. 2016), were grown in a greenhouse at 26 ± 2 °C, with a photoperiod of 16 h and relative humidity of 85% to 100%, as previously reported (Feng et al. 2016). The bacterial strains and plasmids used in this study are listed in Table 1. To generate the *OsPGIP1* overexpression transgenic lines, the complete *OsPGIP1* gene was cloned into plasmid pU1301, directly after the maize ubiquitin constitutive promoter via *Kpn* I and *Bam*H I restriction sites to make pU1301::OsPGIP1. To generate the *OsPGIP1*-silenced rice, an *OsPGIP1* gene fragment 570 bp in size was inserted into pDS1301 using *Kpn* I and *Bam*H I, and a second inverted fragment was inserted using *Sac* I and *Spe* I to generate ds1301::OsPGIP1, which mediated RNA interference by expressing double-stranded RNA in rice (Li et al. 2013). The recombinant plasmids of pU1301::OsPGIP1 and ds1301::OsPGIP1 were introduced into *A. tumefaciens* strain EHA105.

**Table 1:** Bacterial strains and plasmids

Strains/plasmids	Characteristics	Source
Strains		
DH5α	*Escherichia coli* for plasmids transformation	Lab collection
EHA105	*Agrobacterium tumefaciens* for rice transformation, Rif^R^	Lab collection
RS105	Wild type* Xoc* strain, Rif^R^	Zou et al. 2012
RS105Δpg	Deletion of *XocPG* gene in RS105, Rif^R^	This study
RS105Δpg-CP	*XocPG* gene complementation in RS105Δpg, Km^R^, Rif^R^	This study
Plasmids		
pK18mobsacB	Suicide vector for homologous recombination, Km^R^	Schäfer et al. 1994
pK18mobsacB-PG	Deletion, upstream and downstream fragments of *XocPG* cloned in suicide vector pK18mobsacB, Km^R^	This study
pVSP61	Expression vector, Km^R^	Loper and Lindow 1987
pVSP61-PG	Complementation, *XocPG* cloned in pVSP61 vector, Km^R^	This study
pU1301::OsPGIP1	Rice transformation, *OsPGIP1* cloned in constitutive expression vector pU1301, *Ubi*, Km^R^	This study
pDS1301::OsPGIP1	Rice transformation, *OsPGIP1* fragment cloned in RNA-silenced vector pDS1301, *35S*, Km^R^	This study
BD-PG	Yeast two-hybrid, *XocPG* cloned in the bait vector pGBKT7, Km^R^	This study
AD-OsPGIP1	Yeast two-hybrid, *OsPGIP1* cloned in the prey vector pGADT7, Amp^R^	This study
AD-OsPGIP4	Yeast two-hybrid, *OsPGIP4* cloned in the prey vector pGADT7, Amp^R^	This study
pGBKT7–53	Yeast two-hybrid, positive bait vector with murine p53, Km^R^	Clontech
pGADT7-T	Yeast two-hybrid, positive bait vector with SV40 large T-antigen, Amp^R^	Clontech

### Gene Expression Analysis

Infected leaves were collected at 0, 2, 4, 8, 24, and 96 h postinoculation (hpi) for ZH11 and at 0, 6, 24, 48, and 96 hpi for Acc8558. Total RNA was extracted using TRIzol reagent (Sigma-Aldrich, Germany). *OsPGIP1* expression was confirmed in different rice varieties (ZH11 and Acc8558) inoculated with RS105 using quantitative PCR (qPCR). First strand cDNA was generated using ReverTra Ace qPCR RT Master Mix with gDNA Remover kit (TOYOBO, Japan). Quantitative real-time PCR was performed on a QuantStudio™ 6 Flex Real-Time System (Applied Biosystems, USA) with KOD SYBR qPCR Mix (TOYOBO). The qPCR program followed that described in Ju et al. 2017, using *OsACTIN* (LOC_Os03g50890) as an internal control (Additional file 2: Table S1).

### Manipulation of the Polygalacturonase Gene in *X. oryzae* pv. *oryzicola*

To generate the knockout mutation of PG in *Xoc*, we used the RS105 putative polygalacturonase sequence (GenBank accession No. WP_014503099) to design the primers XocPG-up and XocPG-down (Additional file 2: Table S1) that amplified a 935 bp fragment upstream and a 963 bp fragment downstream of the gene from RS105. The two fragments were fused into one fragment and then cloned into pK18mobsacB using *Bam*H I and *Hin*d III to generate the plasmid pK18mobsacB-PG. To generate a homologous recombination mutant, we introduced the plasmid pK18mobsacB-PG into RS105 competent cells as previously described (Zou et al. 2012). Single crossover mutants were selected using kanamycin as a selective marker, and colonies that grew were then transferred to sucrose screening medium to select a double exchange unmarked mutant. The RS105Δpg strain resulting from homologous recombination was further confirmed by PCR and DNA sequencing.

To complement RS105Δpg, a 2285 bp genomic DNA fragment that included the promoter and full coding sequence of the protein was amplified using pairs of forward and reverse primers for XocPG-CP (Additional file 2: Table S1). The fragment was double digested with *Eco*R I and *Hin*d III and cloned into pVSP61. The recombined plasmid was transformed into RS105Δpg competent cells to obtain the complement RS105Δpg-CP.

### Pathogen Inoculation Growth Curve and Resistance Assessment

The *Xoc* strains RS105, RS105Δpg and RS105Δpg-CP were grown on polypeptone-sucrose-agar medium (10 g l^− 1^ polypeptone, 1 g l^− 1^ glutamic acid, 10 g l^− 1^ sucrose and 15 g l^− 1^ agar) at 28 °C for 3 days and then resuspended in sterile water to an OD_600_ of 0.5 (Ju et al. 2017). Fully expanded leaves of four- to six-week-old rice were inoculated with a bacterial suspension of RS105, RS105Δpg or RS105Δpg-CP using a blunt end syringe. Disease severity was assessed by measuring water-soaked lesion length at 14 days postinoculation (dpi). To investigate the *in planta* bacterial growth, rice leaves were inoculated with a bacterial suspension of a *Xoc* strain, RS105, RS105Δpg or RS105Δpg-CP, at an OD_600_:0.1 of. Inoculated leaves were harvested and used for determining bacterial populations at 1, 4, and 7 dpi as previously described (Ju et al. 2017; Yang et al. 2019).

### Yeast Two Hybrid

The 1572 bp DNA fragment of the *PG* gene that deleted the transmembrane domain was amplified from the RS105 genome using pairs of XocPG-BD primers (Additional file 2: Table S1) and then cloned into pGBKT7 as a bait vector BD-PG. The 870 bp DNA fragment of *OsPGIP1* and 968 bp DNA fragment of *OsPGIP4* that deleted the transmembrane domain were amplified using forward and reverse primers of OsPGIP1-AD and OsPGIP4-AD, respectively (Additional file 2: Table S1), from ZH11 cDNA and then cloned into pGADT7 as the prey vectors AD-OsPGIP1 and AD-OsPGIP4. The BD-PG and AD-OsPGIP1 vectors, and BD-PG and AD-OsPGIP4 vectors were cotransformed into Y2H golden yeast cells through PEG-LiAc-mediated transformation according to the instructions of the Yeastmaker Yeast Transformation System 2 (Clontech, the USA). The yeast transformants grew on double-dropout minimal base (SD/−leucine-tryptophan), and the interaction in yeast was tested by quadruple-dropout minimal base (SD/−leu-trp-ade-his) and aureobasidin A. The pGBKT7–53 and pGADT7-T vectors were used as positive controls in the yeast transformation protocol.

### RNA-Seq and Analysis

As previously described (Ju et al. 2017; Zhang et al. 2018), mixed RNA samples of noninfected and infected (24 hpi) leaves of ZH11 and OV-24 were used for library construction and sequencing with BGISEQ-500 by the Beijing Genomic Institution (www.genomics.org.cn, BGI, Shenzhen, China). In brief, 9 individuals of ZH11 and OV-24 were grown side-by-side in one container. After 6 weeks of growth, the rice leaves were inoculated with RS105 and collected for RNA extraction at 0 hpi and 24 hpi. For each sample, we collected approximately 100 mg of leaves from three individuals to extract total RNA with TRI reagent to form three replicates for ZH11, ZH11-RS (ZH11 inoculated with RS105 at 24 hpi), OV-24 and OV-24- RS (OV-24 inoculated with RS105 at 24 hpi). Subsequently, equal amounts of total RNA from three replicates were mixed together for library construction. The above experiments were repeated again, and the sequencing reads were collected and analyzed separately. The clean reads were aligned to the rice Nipponbare reference genome (http://rice.plantbiology.msu.edu). Gene expression levels were quantified via fragments per kilobase of exon per million fragments mapped (FPKM). Genes with a *P*-value< 0.001 and log2 (FPKM-*OV-24*/FPKM-ZH11 or FPKM- ZH11-RS/FPKM-ZH11 or FPKM-*OV-24-*RS/FPKM-*OV-24*) > 1 were considered differentially expressed genes (DEGs). DEGs commonly repeated in two experiments were collected for further functional analysis. Gene Ontology (GO) analysis of the DEGs in the GO database (http://www.geneontology.org/) was used to recognize the main biological functions. The two transcriptome datasets have been deposited in the NCBI Sequence Read Archive Database (http://trace.ncbi.nlm.nih.gov/Traces/sra) under the Accession Number PRJNA517024.

### Hormone Determination and Treatment

ZH11 and *OsPGIP1*-overexpressing rice were grown for 6 weeks in a greenhouse and then inoculated with RS105. Inoculated and noninoculated leaves with RS105 at 24 hpi were collected separately and prepared for hormone quantification. Three biological replicates of approximately 100 mg to 150 mg leaves were used to measure the contents of SA and JA according to a previous reference (Xu et al. 2016).

### Statistical Analysis

Each experiment was repeated at least three times independently. Statistical analyses were performed with SPSS software. Student’s *t-*test and least significant difference (LSD) test were used for significant analysis, and a *P* test value less than 0.05 was considered significant.

## Results

### The Expression Patterns of Seven *OsPGIPs* after Inoculation with *Xoc* RS105

**Fig. 1:**
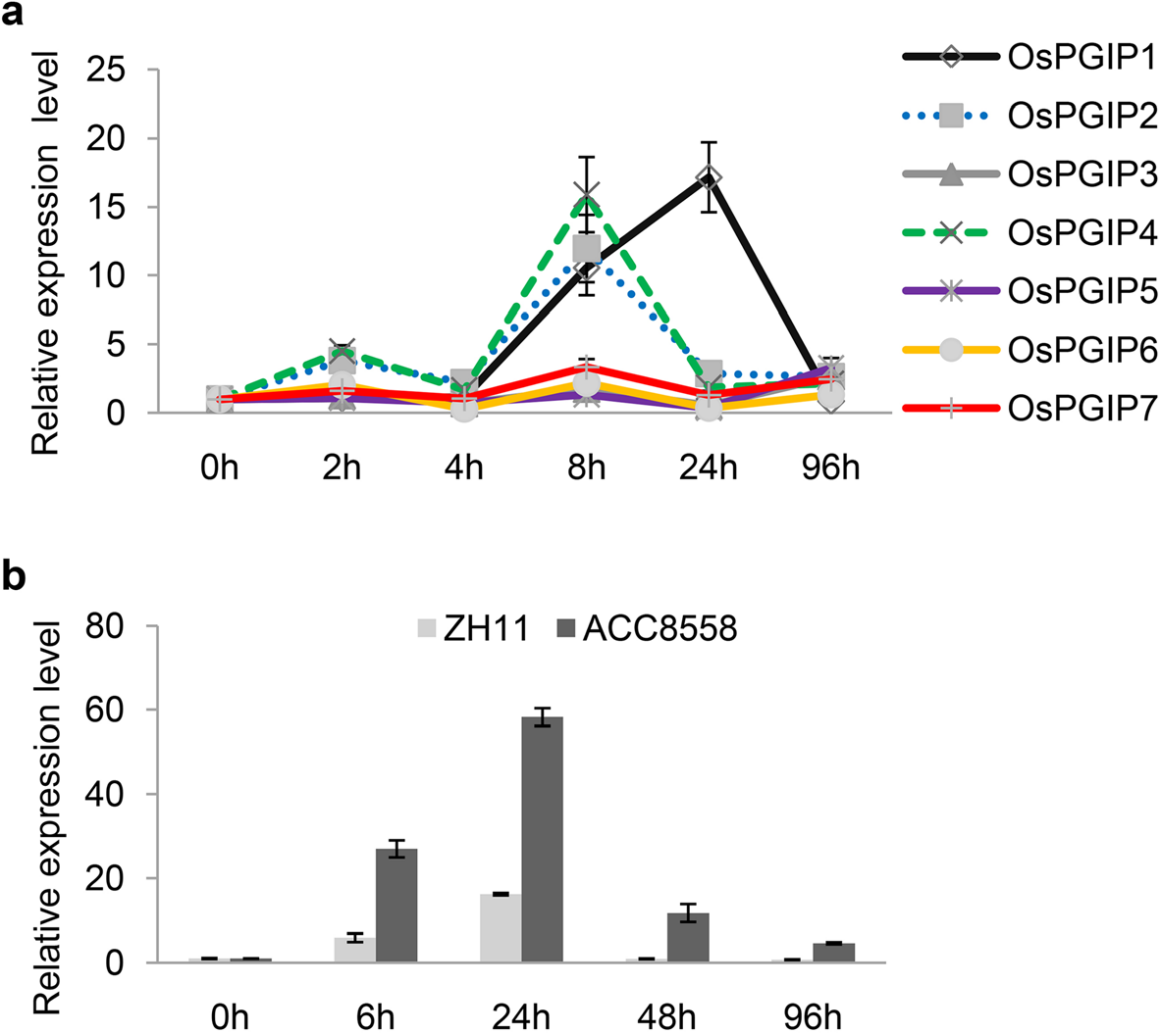
Expression patterns of rice *OsPGIPs* in response to the *Xanthomonas oryzae* pv. *oryzicola* strain RS105. **a** Relative expression of *OsPGIPs* in ZH11 in response to *Xoc*. The gene expression levels of *OsPGIP1* to *OsPGIP7* were analyzed by qRT-PCR after inoculation with *Xoc* stain RS105 at 2, 4, 8, 24, and 96 h. **b** The expression of *OsPGIP1* in response to RS105 at 6, 24, 48, and 96 h in the susceptible rice variety ZH11 and moderately resistant variety Acc8558. The housekeeping gene *ACTIN* was used to normalize the data. Error bars represent the standard deviations for three replicates. Three independent experiments were performed with the same expression pattern

There are seven *OsPGIPs* (*OsPGIP1–7*) throughout the genome of rice, of which the expression of five *OsPGIPs*, excluding *OsPGIP6* and *OsPGIP7*, was strongly induced after inoculation with the fungal pathogen *R. solani* (Lu et al. 2012). However, the response of *OsPGIPs* to the bacterial pathogen *Xoc* remains unclear. Thus, we detected the expression of all *OsPGIP* members during infection with the *Xoc *strain RS105. The relative expression of *OsPGIP1* was continuously increased during the initial 24 h after inoculation and then rapidly decreased to normal levels from 24 hpi to 96 hpi (Fig. 1a). In contrast to *OsPGIP1*, six other *OsPGIPs* showed decreased expression at 4 hpi (Fig. 1a), indicating a unique function of *OsPGIP1* in BLS resistance. Moreover, *OsPGIP2* and *OsPGIP4* had showed the most similar expression patterns during *Xoc* infection, which presented a peak expression level at 8 hpi, and the expression patterns of *OsPGIP3* and *OsPGIP5* and of *OsPGIP6* and *OsPGIP7* were similar to each other (Fig. 1a). We further investigated the difference in the expression of *OsPGIP1* to *OsPGIP7* between the susceptible variety ZH11 and the moderately resistant variety Acc8558. *OsPGIP1* had a similar expression pattern but was induced at higher levels in Acc8558 than in ZH11 (Fig. 1b). In addition, the expression patterns of *OsPGIP2* and *OsPGIP4* after inoculation with RS105 in Acc8558 were also similar to those in ZH11, but the expression levels were even higher in Acc8558 (Additional file 7: Figure S1). Then, we analyzed the promoter sequence of *OsPGIP1*, which showed several differences between ZH11 and Acc8558 (Additional file 1: Text S1). The promoter sequence of *OsPGIP1* in Acc8558 contains more ethylene-responsive elements (ERE-motif) and SEB-1 binding sites (STRE-motif) but one less W-box, GATA-motif and Myc, which may respond to the higher induction of *OsPGIP1* in Acc8558, than that of ZH11 (Additional file 3: Table S2). Overall, the expression pattern of *OsPGIP1* upon inoculation with RS105 implied that it may be involved in resistance to *Xoc*.

### *OsPGIP1* Contributes to Bacterial Leaf Streak Resistance

**Fig. 2:**
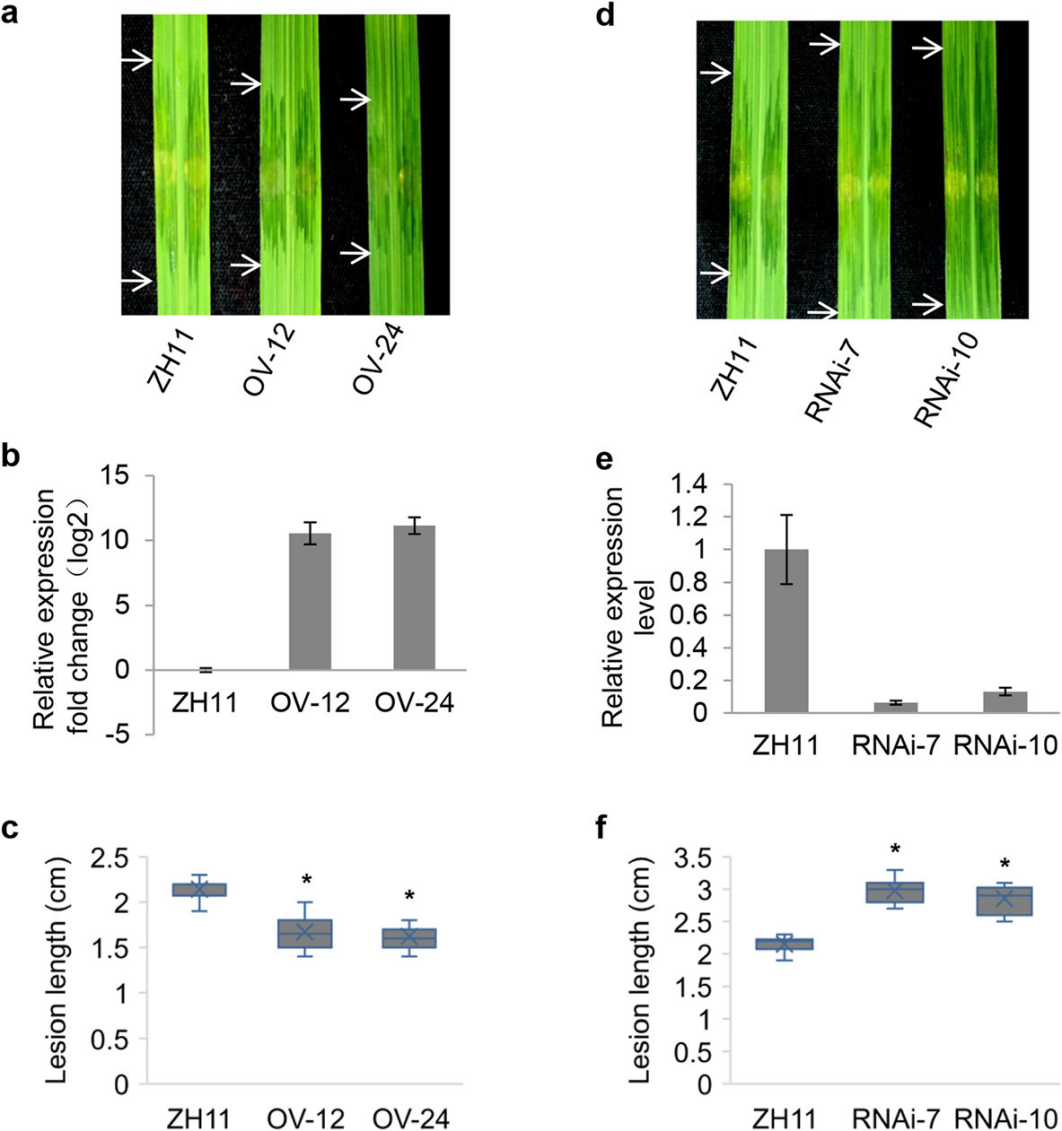
*OsPGIP1* contributes to bacterial leaf streak resistance. **a** The phenotypes of the RS105 lesions that developed on ZH11 and two *OsPGIP1*-overexpressing lines (OV-12 and OV-24) at 14 days after infiltration. The arrows represent the boundaries of lesion expansion caused by RS105 infection. **b** Relative expression fold change of *OsPGIP1* in OV-12 and OV-24. The expression of wild-type ZH11 was used as a control and set to 1. The housekeeping gene *ACTIN* was used to normalize the data. **c** The lesion lengths of ZH11, OV-12 and OV-24 at 14 days after inoculation with RS105. **d** The phenotypes of the RS105 lesions that developed on ZH11 and two *OsPGIP1*-suppressed lines (RNAi-7 and RNAi-10) at 14 days after infiltration. The arrows represent the boundaries of lesion expansion caused by RS105 infection. **e** Relative expression level of *OsPGIP1* in RNAi-7, RNAi-10 and ZH11. The expression of wild-type ZH11 was used as a control and set to 1. *ACTIN* was an internal reference gene for normalization. **f** The lesion lengths of ZH11, RNAi-7 and RNAi-10 inoculated with RS105 after 14 days. Data were analyzed using a *t*-test. Asterisks represent statistically significant differences from the ZH11 wild type at *P* < 0.05

To verify that *OsPGIP1* is involved in BLS resistance, we generated *OsPGIP1-*overexpressing and *OsPGIP1-*silenced transgenic lines of rice in susceptible ZH11 by *A. tumefaciens*-mediated transformation. A total of 29 *OsPGIP1-*overexpressing transgenic lines were generated in the T_0_ generation. Twelve of them were confirmed using the specific primers Hpt-Forward and Hpt-Reverse (Additional file 2: Table S1). Compared to the wild-type ZH11, these same 12 lines showed significantly shorter lesion lengths than after inoculation with RS105 (Additional file 8: Figure S2a). Based on the lesion length scored in the T_0_ generation, three lines were selected for further characterization in the T_1_ generation. This included the moderately resistant line pU1301::OsPGIP1–12 (1.625 ± 0.176 cm), the most resistant line pU1301::OsPGIP1–24 (1.505 ± 0.165 cm), and the weakest resistant line pU1301::OsPGIP1–29 (1.825 ± 0.226 cm) (named OV-12, OV-24 and OV-29, respectively, hereafter). As shown in Additional file 8: Figure S2b, the resistance phenotype is cosegregated with pU1301::OsPGIP1, as identified by DNA amplification in all three T_1_ progeny. We also observed the resistance phenotype to RS105 in the OV-12 and OV-24 lines in the T_2_ progeny (Fig. 2a). The relative expression level of *OsPGIP1* in these lines showed approximately 1500-fold and 2000-fold enhanced expression in the OV-12 and OV-24 lines compared with that in ZH11 (Fig. 2b). Both the increased expression of *OsPGIP1* and shortened lesion length were detected in OV-12 and OV-24 (Fig. 2a, c). Alternatively, OV-24 had increased resistance to RS105 compared to OV-12, and the expression level of *OsPGIP1* was higher in OV-24 than in OV-12 (Fig. 2b, c). In addition, the bacterial population of RS105 in the *OsPGIP1*-overexpressing line OV-24 was significantly reduced compared to ZH11 (Additional file 11: Figure S5). Together, these data suggest that the overexpression of *OsPGIP1* enhanced the resistance of rice to *Xoc*.

Three independent silenced transgenic lines were generated, RNAi-7, RNAi-10 and RNAi-11. These three lines were inoculated with RS105 in the T_1_ progeny. We observed cosegregation in these three lines, and all lines were verified as ds1301::OsPGIP1 using primers Hpt-Forward/Reverse by PCR. Furthermore, these two silenced lines were more susceptible to RS105, indicated by longer lesion lengths when compared to WT ZH11 (Additional file 9: Figure S3). Additional studies in the T_2_ generation of silenced plants, RNAi-7 and RNAi-10, demonstrated that *OsPGIP1* mRNA was reduced in RNAi-7 and RNAi-10 by 94% and 87% compared to that in WT, respectively (Fig. 2e). The lesion length reached an average of 3.06 ± 0.26 cm and 2.84 ± 0.20 cm compared to 2.17 ± 0.10 cm in ZH11 (Fig. 2d, f). We also found that the RS105 population in the *OsPGIP1*-silenced line RNAi-10 was larger than that in ZH11 (Additional file 11: Figure S5). These results suggest that silencing the expression of *OsPGIP1* enhanced the susceptibility of rice to BLS.

*OsPGIP1* is located in the same locus as the QTL *qBlsr5a* in the moderately resistant variety Acc8558 (Xie et al. 2014), implying that it may play a role in *qBlsr5a*-mediated resistance. To test this, we generated *OsPGIP1* RNAi lines*.* The three individual RNAi lines of the T_1_ generation in the Acc8558 background were more susceptible to RS105 than wild-type Acc8558 (Additional file 10: Figure S4), which supports that *OsPGIP1* is a defense-related gene that contributes to BLS resistance.

### A PG Works as a Facilitator of the Pathogenic Function in *Xoc*

**Fig. 3:**
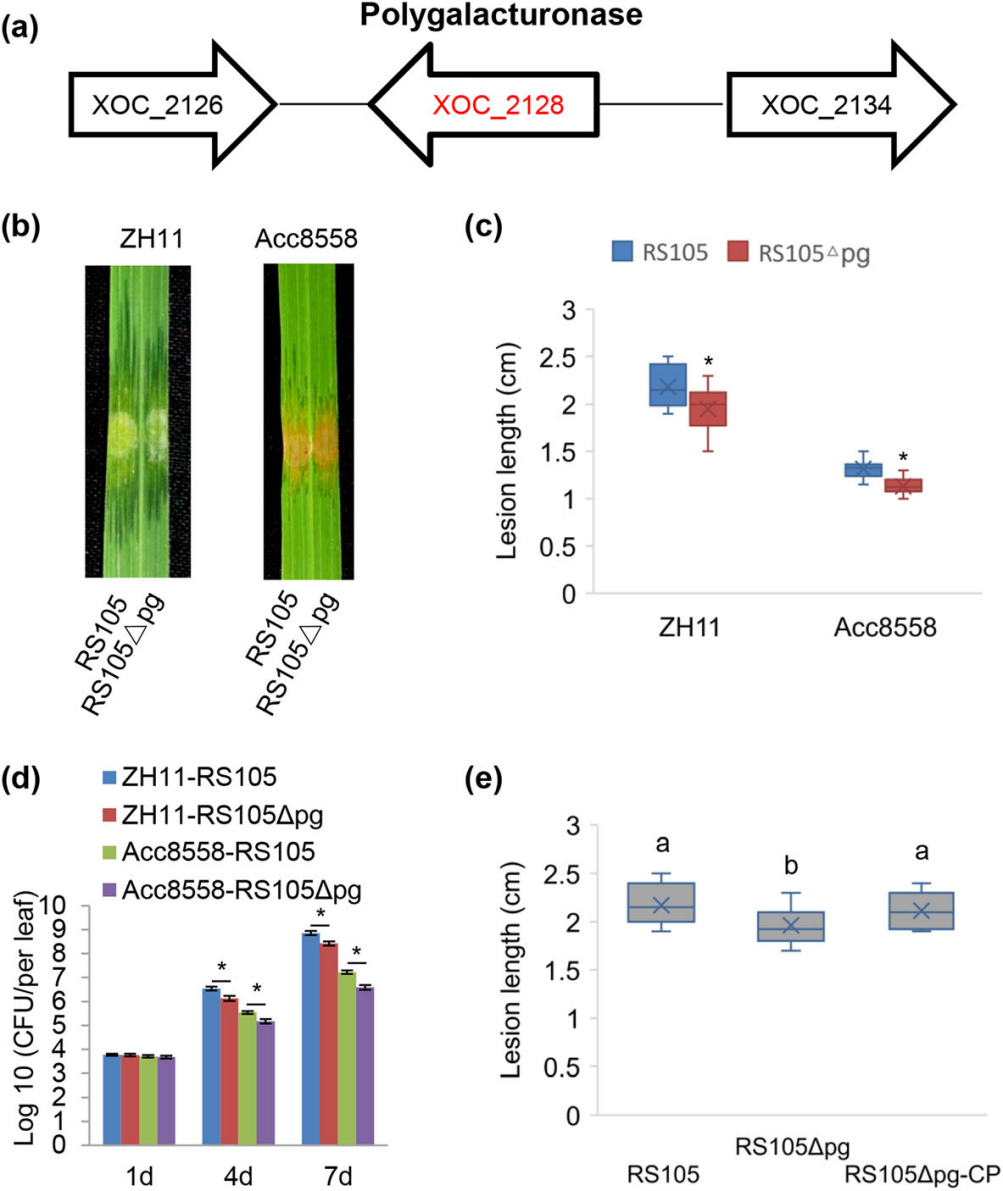
The polygalacturonase gene acts as a virulence factor of RS105 during infection in rice. **a** The position of the polygalacturonase (*PG*) gene in the *Xoc* RS105 genome. **b** The phenotype of RS105 and the *PG* mutant strain (RS105Δpg) on the susceptible rice variety ZH11 and moderately resistant variety Acc8558. Images were photographed 14 days after inoculation. **c** The statistical counting of lesion length at 14 days after inoculation with RS105 and RS105Δpg on ZH11 and Acc8558. The data were counted from over 10 plants and analyzed using a *t*-test (*P* < 0.05). Asterisks represent statistically significant differences from ZH11. **d** The bacterial growth curves of RS105 and RS105Δpg in ZH11 and Acc8558 rice at 1, 4, and 7 days. Significant differences were determined by *t* test: *P < 0.05. **e** Lesion length after inoculation with RS105, RS105Δpg and *PG* gene complementary stain (RS105Δpg-CP) at 14 days in ZH11. The letters above the bars represent the significant differences at a value of *P* ≤ 0.05 (LSD test). The above experiments were repeated three times with similar results

Fungal PGs have been reported as virulence factors in *Botrytis cinerea* (ten Have et al. 1998) and *Claviceps purpurea* (Oeser et al. 2002). To determine whether PGs act as virulence factors in the bacteria *Xoc*, we generated the *PG* gene mutant of RS105Δpg by DNA recombination. The lesion length caused by RS105Δpg was shorter than that cause by RS105 in both the susceptible rice ZH11 and moderately resistant rice Acc8558 (Fig. 3b, c). Furthermore, we also performed a bacterial growth curve *in planta* of RS105 and RS105Δpg, which revealed that the populations of RS105Δpg were less than those of RS105 in both ZH11 and Acc8558 (Fig. 3d). To verify that the PG mutant was the cause of the reduced virulence, we constructed the PG complementation strain RS105Δpg-CP. Inserting a copy of the wild-type gene nto the mutant strain RS105Δpg restored the lesion lengths to the wild-type lesion lengths (2.12 ± 0.18 cm), similar to wild-type RS105 in ZH11 (2.16 ± 0.21 cm) (Fig. 3e). Thus, we conclude that PG acts as a pathogenicity factor in *Xoc* strain RS105.

### *OsPGIP1*-Mediated Resistance Is Induced by Other Pathogenicity Factors in Addition to PG

**Fig. 4:**
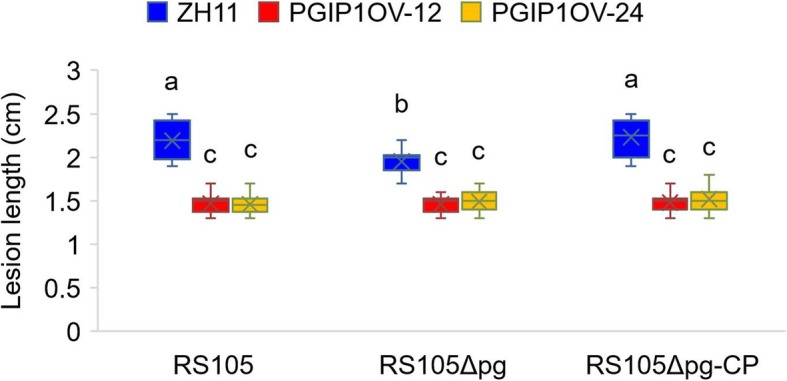
*OsPGIP1*-mediated resistance is complemented and induced by XocPG in addition to other *Xoc* pathogenicity factors. Lesion lengths of RS105, RS105Δpg and RS105Δpg-CP in ZH11 and *OsPGIP1*-overexpressing lines OV-12 and OV-24. The letters above the bars represent the significant differences at a value of *P* ≤ 0.05 (LSD test). The above experiment was repeated three times with similar results

Previous studies demonstrated that PGIPs directly interact with fungal PGs and inhibit the PG enzyme activities that initiate OG-mediated PTI to restrict pathogen growth (Wang et al. 2015a, 2018; Benedetti et al. 2015). Although we have found that the overexpression of *OsPGIP4* enhances resistance to *Xoc* (Feng et al. 2016), the role of OsPGIP1 in response to plant pathogenic bacteria remains unclear. To explore the possibility of direct protein-protein interactions between OsPGIP1and PG, we conducted a yeast two-hybridization experiment. No protein-protein interactions were observed under the conditions tested (Additional file 12: Figure S6). We identified PG as a pathogenicity factor in rice-*Xoc* interactions (Fig. 3b, c and d), and the overexpression of *OsPGIP1* (Fig. 2a, b and c) increased resistance to *Xoc*. We questioned whether *OsPGIP1* resistance was mediated in response to PG alone or if there were additional pathogen-associated molecular patterns that were perceived by this gene. To test this hypothesis, we measured the lesion lengths of ZH11, OsPGIP1 OV-12 and OsPGIP1 OV-24 caused by RS105, RS105Δpg and RS105Δpg-CP. RS105Δpg caused shorter lesions in ZH11 compared to RS105 and RS105Δpg-CP and caused similar lesion lengths in the two *OsPGIP1* OV lines (Fig. 4). This indicates that the PG-dependent virulence was completely abolished by the overexpression of *OsPGIP1*. Moreover, compared to ZH11, in the *OsPGIP1* OV lines, resistance to RS105Δpg was demonstrated (Fig. 4), indicating that *OsPGIP1-*mediated resistance is not only responsive to the PG in *Xoc* but also responsive to other virulence factors in *Xoc* or *Xoc*-induced susceptible factors in rice.

### RNA-Seq Analysis of *OsPGIP1*-Overexpressing Rice

**Fig. 5:**
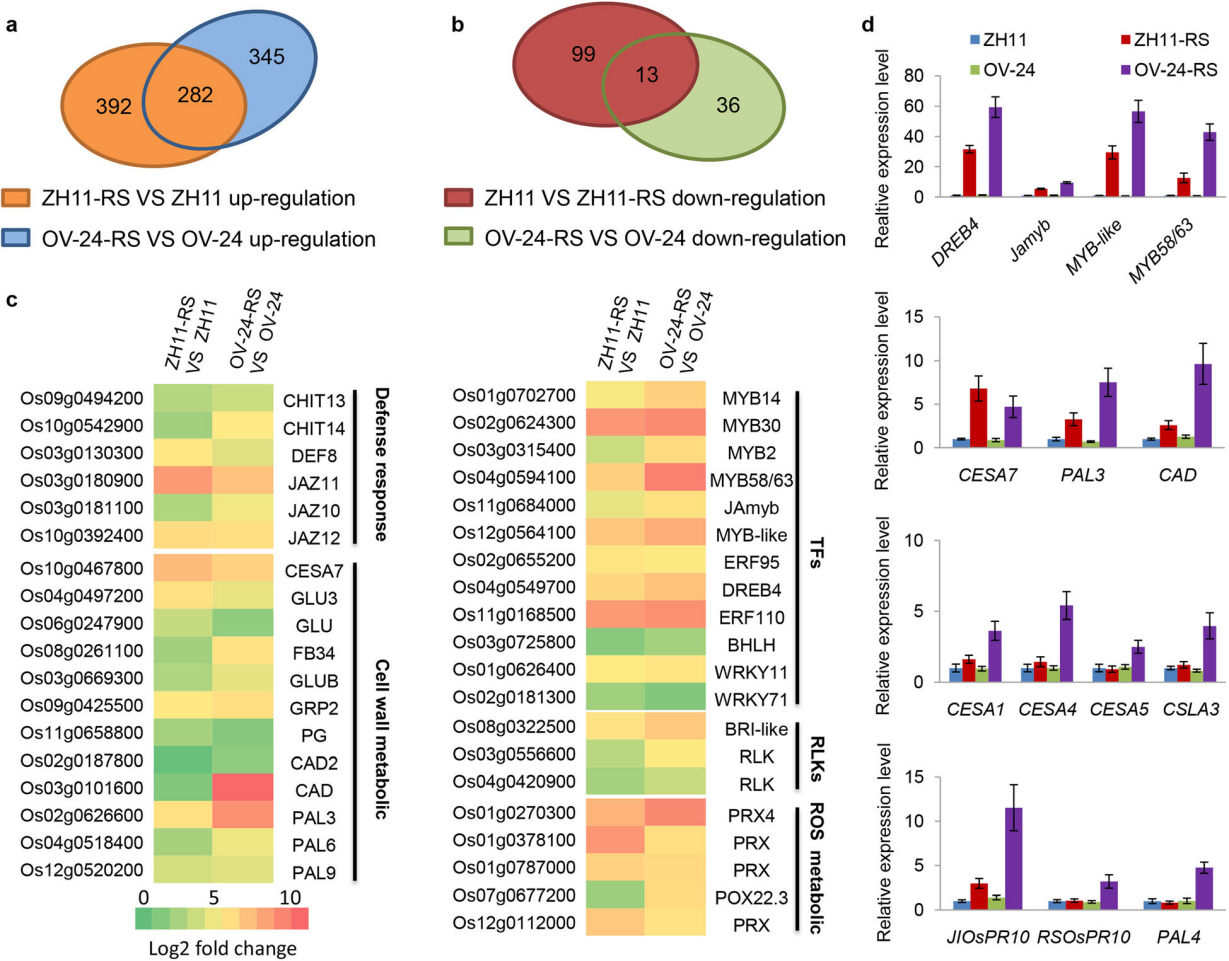
RNA-seq analysis of differentially expressed genes between the *OsPGIP1-*overexpressing line (OV-24) and ZH11 in response to RS105 inoculation. **a** and **b** Venn diagram of upregulated (**a**) or downregulated (**b**) DEGs in response to RS105 at 24 h postinoculation. DEGs were considered at *P* value< 0.001 and |log2-fold change| > 1. ZH11-RS vs. ZH11 and OV-24-RS vs. OV-24 represent the common DEGs identified in two experimental repeats, with noninfected ZH11 and OV-24 as controls. **c** Heatmap analysis of the common significantly upregulated DEGs in OV-24-RS vs. OV-24 and ZH11-RS vs. ZH11. The heatmap was classified into defense response, cell wall metabolism, transcription factors (TFs), receptor-like kinases (RLKs) and ROS metabolism. The intensity of color indicates the expression level of genes from left to right, indicating higher gene expression. **d** The expression of the transcription factor genes, cell wall-related genes and defense response genes that were upregulated in RNA-seq analysis was evaluated by qRT-PCR in ZH11 and OV-24. The internal control gene was *ACTIN.* The above experiments were repeated three times with similar results

To identify the genes that contribute to *OsPGIP1*-mediated resistance to *Xoc*, we performed transcriptome sequencing analysis in the *OsPGIP1*-overexpressing line OV-24 (T_3_ generation) and wild type ZH11. We found that only 138 DEGs, including 75 upregulated and 63 downregulated genes, were differentially expressed in OV-24 compared with ZH11 (Additional file 13: Figure S7a). Of the 138 DEGs, only 3 were defense-response genes, and most were predicted to be of unknown function (Additional file 13: Figure S7a). Upon inoculation with RS105, 786 and 676 DEGs were identified in ZH11-RS and OV-24-RS compared with ZH11 and OV-24, respectively. The DEGs of ZH11-RS vs. ZH11 contained 674 upregulated genes and 112 downregulated genes, while the DEGs of OV-24-RS vs. OV-24 contained 627 upregulated genes and 49 downregulated genes (Fig. 5a, b). The DEGs of ZH11-RS vs. ZH11 and OV-24-RS vs. OV-24 showed that 297 genes were commonly differentially expressed; 379 DEGs specifically responded to *Xoc* in OV-24-RS vs. OV-24, and 489 DEGs changed only in ZH11-RS vs. ZH11 (Additional file 13: Figure S7b). Among the 297 common DEGs, 282 and only 13 genes were upregulated and downregulated, respectively (Fig. 5a, b and Additional file 4: Table S3). Comparisons of the 138 DEGs of OV-24 vs. ZH11 with ZH11-RS vs. ZH11 or OV-24-RS vs. OV-24, 30 and 32 common DEGs were identified, respectively, and only 15 were commonly shared for both (Additional file 13: Figure S7b). More DEGs were identified after inoculation with *Xoc*, indicating that *OsPGIP1*-mediated resistance relied on enhanced gene expression upon *Xoc* infection.

### Functional Analysis of the Three Categories of DEGs in Response to *Xoc* in *OsPGIP1*-Overexpressing Rice

The results showed that 282 DEGs were upregulated in both ZH11-RS and OV-24-RS (Fig. 5a, b). A heatmap representing the analysis of the upregulated genes is shown in Fig. 5c. The GO functions of the 282 upregulated DEGs in both OV-24-RS and ZH11-RS were classified into five categories: defense response, cell wall metabolism, transcription factors, receptor-like kinases (RLKs) and ROS related (Fig. 5c). Of the 282 commonly upregulated DEGs, 143 genes showed higher expression in OV-24-RS than in ZH11-RS (Additional file 4: Table S3). Of the 345 upregulated and 34 downregulated genes specifically regulated in OV-24-RS vs. OV-24, we identified 5 genes related to defense response, 32 genes related to cell wall metabolism, 10 genes related to polysaccharide metabolism, 6 genes related to chitin catabolism and 41 genes related to oxidation-reduction (Additional file 5: Table S4 and Additional file 14: Figure S8).

It has previously been shown that combining the expression of the pathogenesis-related (*PR*) genes of chitinase and β-1,3-glucanase enhanced the resistance of rice to *R. solani* (Sridevi et al. 2008). *CHIT13* (*Os09g0494200*) and *CHIT14* (*Os10g0542900*), which encode chitinases, were significantly induced in OV-24 compared to ZH11 (Fig. 5c). The *PR* genes *JIOsPR10* (*Os03g0300400*), *RSOsPR10* (*Os12g0555000*) and *JAmyb* (*Os11g0684000*) respond to *Magnaporthe grisea* inoculation and JA treatment (Jwa et al. 2001; Hashimoto et al. 2004; Cao et al. 2015). These three *PR* genes had increased expression in OV-24 compared with ZH11 (Fig. 5d and Additional file 5: Table S4). *PAL4* (*Os02g0627100*) is a positive regulator in rice broad-spectrum disease resistance (Tonnessen et al. 2015), and it was also induced in OV-24 (Fig. 5d). Cell wall metabolism is particularly involved in the biotic stress response (Le Gall et al. 2015). We also observed that the cell wall biosynthesis genes *CESA7* (*Os10g0467800*), *GLU* (Os04g0497200) and *GLUB* (*Os03g0669300*) were induced in OV-24 and ZH11 (Fig. 5c). Lignin is a main complex of phenolic polymers that exist in plant cell walls and affects defense signaling in biotic stress (Gallego-Giraldo et al. 2018). We identified that a series of lignin biosynthesis genes were more highly expressed in OV-24 than in ZH11, including *PAL3* (*Os02g0626600*), *PAL6* (*Os04g0518400*), *PAL7* (*Os05g0427400*) and *CAD* (*Os03g0101600*), which were also exhibited expression upregulated by qRT-PCR (Fig. 5c, d). Additionally, the cellulose synthase genes *CESA1* (*Os05g0176100*), *CESA4* (*Os01g0750300*), *CESA5* (*Os03g0837100*), and *CSLA3* (*Os06g0230100*) and lignin synthesis gene *PAL3* (*Os02g0626100*) were upregulated only in OV-24 in addition to the common cell wall biosynthesis genes in OV-24 and ZH11 (Fig. 5c, d). In conclusion, *OsPGIP1*-mediated resistance to *Xoc* may rely on activating the expression of *PR* genes and cell wall-responsive genes.

### Overexpression of *OsPGIP1* or *OsPGIP4* Caused the Accumulation of Jasmonic Acid Postinoculation with *Xoc*

**Fig. 6:**
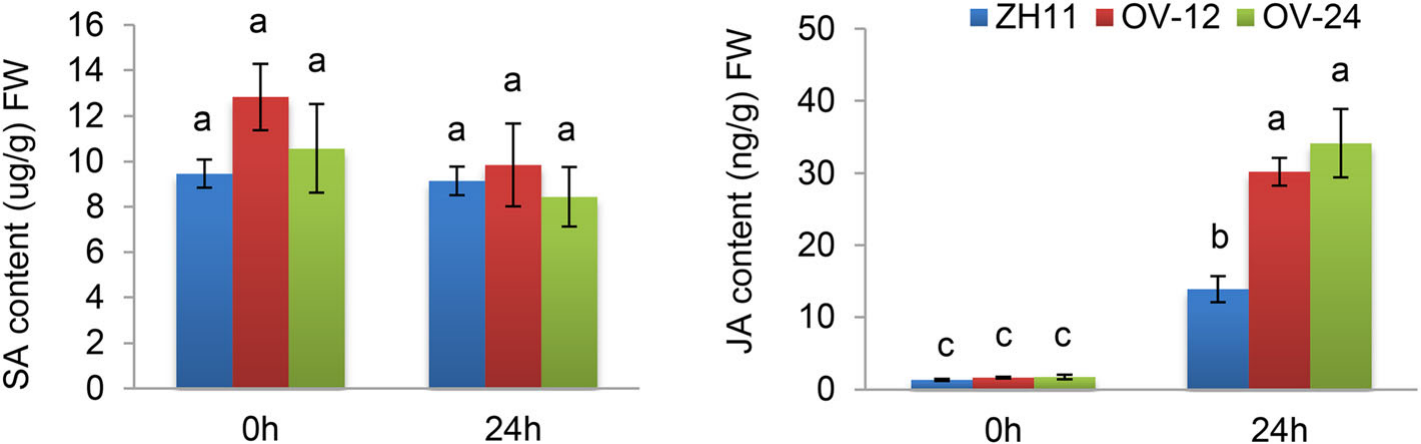
Increased accumulation of jasmonic acid in *OsPGIP1*-overexpressing compared with ZH11 after inoculation with *Xoc*. The SA and JA contents were measured in the ZH11 and *OsPGIP1*-overexpressing lines OV-12 and OV-24 without inoculation and 24 h postinoculation with RS105. The letters above the bars represent the significant differences at a value of *P* ≤ 0.05 (LSD test)

Generally, hormones in host plants, such as SA, JA and ET, change in response to pathogen attack (Spoel and Dong 2008). The interaction between PGs and PGIPs is associated with the accumulation of OGs inducing host resistance through the hormone pathway (Benedetti et al. 2015; Wang et al. 2018). When the *Fusarium phyllophilum* FpPG and its cognate *Phaseolus vulgaris* PvPGIP2 were ectopically expressed in *Arabidopsis*, the accumulation of excessive SA was detected, which in turn activated the immune response (Benedetti et al. 2015). Our previous studies found that the overexpression of *OsPGIP4* induced the expression of JA biosynthesis-related genes after inoculation with RS105 (Feng et al. 2016). In this study, we found that some JA-related genes were upregulated in OV-24. To determine whether JA is involved in *OsPGIP1-*mediated immunity to *Xoc*, we measured the JA and SA levels in both *OsPGIP1-OV* and wild-type ZH11. The SA levels were not significantly different between the control and RS105-inoculated ZH11, OV-12 and OV-24 rice plants (Fig. 6). In the noninoculated control plants, the JA levels were similar in ZH11 and two tested *OsPGIP1-*OV rice lines. In the RS105-inoculated plants, the JA level increased in both the ZH11 and *OsPGIP1*-OV rice lines at 24 hpi (Fig. 6). More importantly, the RS105-inoculated plants showed two-fold higher levels of JA in *OsPGIP1* OV rice lines than in ZH11 (Fig. 6). In general, the *OsPGIP1* OV rice lines accumulate more JA to enhance the resistance to *Xoc*.

## Discussion

PGIPs have clearly been shown to protect plants by enhancing their resistance to fungi; however, there are few examples of PGIPs enhancing plant resistance to bacterial pathogens (Kalunke et al. 2015; Feng et al. 2016). Rice has seven *OsPGIPs* genes, and they have been shown to respond to various hormones and fungi (Lu et al. 2012). Previously, the overexpression of *OsPGIP1* was shown to enhance the resistance of rice to *R. solani* (Wang et al. 2015a, b; Chen et al. 2016). Constitutive heterogeneous expression of *OsPGIP2* enhanced the resistance of *B. napus* to *S. sclerotiorum* (Wang et al. 2018). In the present study, we identified that the overexpression of *OsPGIP1* enhanced the resistance of rice, while in contrast, the RNAi rice lines showed decreased resistance to *Xoc* strain RS105 in the susceptible variety ZH11 (Fig. 2). Additionally, repressing *OsPGIP1* expression attenuated resistance to RS105 in the moderately resistant variety Acc8558 (Additional file 10: Figure S4). We concluded that *OsPGIP1* contributes to BLS resistance. In addition to *OsPGIP4,* which is located in the closely linked region of *OsPGIP1* on chromosome 5 (Feng et al. 2016), we supplied *OsPGIP1* as an additional example of a PGIP that combats bacterial pathogens in rice.

The interaction between PGIPs and PG plays crucial roles in resistance to pathogens. PvPGIP2 from *P. vulgaris* can inhibit the activity of PGs from *Aspergillus niger*, *Fusarium moniliforme* and *F. phyllophilum* (Leckie et al. 1999; Benedetti et al. 2013), and the crystal structure of chemically cross-linked PvPGIP2-FpPG reveals the interaction of PvPGIP2 and FpPG (Benedetti et al. 2011). Ectopic expression of PvPGIP2-FpPG chimera elicited immune responses in *Arabidopsis* that increased resistance to fungal and bacterial phytopathogens (Benedetti et al. 2015). However, wheat expressing *PvPGIP2* shows no resistance to ergot disease because it loses the ability to inhibit PG activity in *Claviceps purpurea* (Volpi et al. 2013). Thus, PGIP-mediated defense responses require PGIP-PG complex formation. Among the seven PGIPs in rice, the recombinant proteins of OsFOR1 and OsPGIP1 inhibited the activity of PG from *A. niger* and *R. solani*, respectively (Jang et al. 2003; Wang et al. 2015b; Chen et al. 2016). Only OsPGIP2 was identified to interact with both SsPG3 and SsPG6 from *S. sclerotiorum* (Wang et al. 2018). Here, we confirmed that the *PG* gene contributed to the virulence of RS105. Compared with RS105, RS105Δpg had attenuated virulence in both the susceptible rice variety ZH11 and the moderately resistant variety Acc8558 (Fig. 3b). Furthermore, there was no significant difference in the disease phenotype caused by RS105 and RS105Δpg in the *OsPGIP1* OV rice (Fig. 4). The results indicate that PG-mediated virulence deficiency was genetically complemented by overexpressing *OsPGIP1.* However, based on yeast two-hybridization (Additional file 12: Figure S6), no obvious interaction was found between PG and OsPGIP1, suggesting a different resistance mechanism of OsPGIP1 for the bacterial pathogen *Xoc* and the fungal pathogen *R. solani*.

Three other observations also supported the above difference. First, the *DR* genes were characterized as having upregulated expression upon pathogen inoculation (Kou and Wang 2010). Lu et al. (2012) investigated the expression pattern of *OsPGIP1*, which slowly increased before 12 hpi, rapidly increased from 12 to 60 hpi, and then decreased slowly from 60 to 96 hpi upon inoculation with *R. solani* WH-1. In the same ZH11 rice variety, the expression of *OsPGIP1* rapidly increased from 0 to 24 hpi and then decreased upon inoculation with *Xoc* RS105 (Fig. 1a). Moreover, the activated level of *OsPGIP1* differed between WH-1 and RS105, with approximately 350-fold and 17-fold peaks in their relative transcript levels, respectively. The different expression patterns implied that they have diversified functions in the resistance to pathogens. Second, PGase activity has been identified from the extraction of PG from *R. solani* (Chen et al. 2016). However, in addition to there being no interaction between XocPG and OsPGIP1, whether the prokaryotic expression of XocPG or crude protein extracted from RS105 and RS105Δpg was used, we did not detect clear PGase activity with agar diffusion assays (data not shown). Third, we found that *OsPGIP1* OV lines primed the accumulation of a higher level of JA but not SA upon inoculation with *Xoc* RS105 (Fig. 6). Although we have not quantified the content of JA and SA upon inoculation with *R. solani*, Onda et al. (2018) described that SA treatment enhanced the resistance while JA treatment enhanced the susceptibility of rice to *R. solani*. Combining the findings that *OsPGIP1* OV lines are still resistant to RS105Δpg (Fig. 4), we believe that *OsPGIP1*-mediated resistance has another unclear mechanism in addition to responding to XocPG.

To understand the mechanism by which *OsPGIP1* mediates resistance to *Xoc*, we performed RNA-seq of *OsPGIP1* OV rice. Without RS105 inoculation, the *OsPGIP1* OV rice showed few gene alterations compared with ZH11, and no defense-related genes were identified. This is consistent with the results of the analysis of the JA and SA contents, which showed no significant changes between *OsPGIP1* OV and ZH11 without inoculation (Fig. 6). A large number of genes were differentially expressed in both *OsPGIP1* OV and ZH11 during inoculation (Additional file 13: Figure S7). Among them, two categories, *PR* genes and cell wall-related genes, were enriched among the common and *OsPGIP1* OV-specific DEGs (Fig. 5). Several *CHITs*, *JIOsPR10* (*Os03g0300400*) and *RSOsPR10* (*Os12g0555000*), were differentially expressed in OsPGIP1 OV lines at 24 hpi with RS105 inoculation (Fig. 5). In addition to *PR* genes, the cell wall defense-associated genes and several MYB transcription factors were highly expressed or characteristically induced in *OsPGIP1* OV rice (Fig. 5 and Additional file 14: Figure S8). The plant cell wall provides a native barrier to block the incursion of different pathogens, and its structure is complex with cellulose, callose, pectins, hemicelluloses, lignin and polysaccharides. Cellulose synthases (CESAs), glucan synthase-like (GSLs) enzymes, and xyloglucan endo-transglycosylases/hydrolases (XTHs) induced expression and participated in cell wall reestablishment (Lampugnani et al. 2018; Bacete et al. 2018). In our study, we found that the cell wall-establishing genes, such as *CESA1*, *CESA4*, *CESA5*, *CESA7* and *CSLA3,* and lignin synthesis genes, including *PAL1*, *PAL3*, *PAL6*, *PAL7* and *CAD,* were upregulated in *OsPGIP1* OV rice after inoculation with RS105 (Additional file 5: Table S4 and Additional file 6: Table S5). The results indicated that *CESA4* and *CESA7* are important cellulose synthase genes for controlling cell wall formation (Tanaka et al. 2003; Zhang et al. 2009; Wang et al. 2016). MYB transcription factors play important roles in regulating cell wall biosynthesis in plants. For instance, *Arabidopsis* MYB46 directly targets the promoters of *CESA4*, *CESA7* and *CESA8* to induce the expression of three cellulose synthase genes that regulate secondary cell wall formation (Kim et al. 2013). The heterogeneous expression of the *PdMYB10*/*128 R2R3-MYB* pair in *Populus* increased the fiber cell wall thickness in *Arabidopsis* (Chai et al. 2014). The expression of *EjMYB1* promotes the expression of *EjPAL1*, *Ej4CLs* and *EjCADs* to increase lignin biosynthesis under cold stress in *Eriobotrya japonica* (Xu et al. 2014). Several rice MYBs are involved in regulating cell wall biosynthesis. The *OsMYB103L* gene plays a role in leaf rolling and directly binds the promoters of *CESA4*, *CESA7*, and *CESA9* to regulate gene expression and promote cell wall formation (Yang et al. 2014; Ye et al. 2015). *OsMYB61* directly regulates *CESA* gene expression to participate in cell wall construction (Huang et al. 2015). Here, we identified three upregulated MYB TFs in common DEGs of *OsPGIP1* OV rice and ZH11 after inoculation with RS105, including the *MYB-like* (*Os12g0564100*), *MYB58/63* (*Os04g0594100*) and *Jamyb* genes (Fig. 5c). All three MYB TFs were more strongly induced in *OsPGIP1* OV rice, which coincided with the expression of cell wall-associated genes (Fig. 5). Among them, *MYB58/63* has been shown to be a positive regulator of *OsCESA7* gene expression (Noda et al. 2015). It is possible that *OsPGIP1-*mediated resistance may protect rice against *Xoc* through cell wall reestablishment in addition to inducing *PR* gene expression.

Overall, we identified that the overexpression of *OsPGIP1* could enhance resistance to BLS in addition to SB (Wang et al. 2015a, b; Chen et al. 2016). Currently, SB is one of the most severe diseases in rice that causes the highest prevalence of infection each year. BLS is becoming the major epidemic bacterial disease, spreading rapidly and widely in southern and central China. Both rice BLS and SB resistance are mainly controlled by quantitative trait loci (Xie et al. 2014; Manosalva et al. 2009), increasing the applied range of *OsPGIP1* in disease-resistance breeding. It was previously shown that there is often a resistance cost upon the overexpression of *DR* genes in addition to enhanced disease resistance.For instance, *OsHSP18.0-CI*-overexpressing rice exhibited lower plant height and smaller panicles than wild-type ZH11 rice (Ju et al. 2017). However, overexpressing *OsPGIP1* has no obvious harmful effects on development and agricultural traits in Xudao3 (Chen et al. 2016). Here, compared to ZH11, two *OsPGIP1* OV lines in the ZH11 background also had no effect on yield traits, including tiller number and 1000 seed-grain weight (Additional file 15: Figure S9). Consistent with the lack of significant resistance cost, the SA and JA contents (Fig. 6) and the expression level of *PR* genes (Fig. 5) showed no significant changes between the *OsPGIP1* OV lines and ZH11 without pathogen challenge. Therefore, we conclude that *OsPGIP1* is an ideal candidate to aid in the development of resistant rice.

## Conclusions

Our study revealed that *PG* is a virulence factor in *Xoc*. *OsPGIP1* is an ideal *DR* gene that contributes to BLS resistance in addition to resistance to SB in rice. We also revealed that the *OsPGIP1*-mediated resistance is induced by XocPG and other *Xoc* pathogenicity factors. It is primed by the activated expression of *PR* genes, the cell wall defense-associated genes and their regulators, and an accumulation of JA.

## Supplementary information

**Additional file 1: Text S1.** Comparison of the *OsPGIP1*sequence containing CDS and promoter between ZH11 and Acc8558.

**Additional file 2: Table S1.** The primers used in this study.

**Additional file 3: Table S2.** The putative *cis*-elements of the *OsPGIP1* promoters in ZH11 and Acc8558.

**Additional file 4: Table S3.** RNA-seq analysis of the common DEGs in ZH11-RS vs. ZH11 and OV-24-RS vs. OV-24.

**Additional file 5: Table S4.** RNA-seq analysis of the unique DEGs of OV-24-RS vs. OV-24.

**Additional file 6: Table S5.** RNA-seq analysis of the unique DEGs of ZH11-RS vs. ZH11.

**Additional file 7: Figure S1.** Expression patterns of rice *OsPGIPs* in response to RS105 in Acc8558 rice. The expression of *OsPGIPs* in BLS moderately resistant rice variety Acc8558 at 6, 24, 48, and 96 h postinoculation was related to leaves without inoculation (0 h). The *ACTIN* was used as an internal control. Error bars represent the standard deviations for three replicates.

**Additional file 8: Figure S2.** Resistance of the *OsPGIP1*-overexpressing plants to the *Xoc* strain RS105 in the T_0_ and T_1_ generation. (a) Lesion length analysis of *OsPGIP1*-overexpressing transgenic rice in the T_0_ generation in a ZH11 background 14 days after inoculation with RS105. (b) Cosegregation of the lesion length with PCR positive selection in the T_1_ generation for the OV-12, OV-24 and OV-29 lines. The average lesion length was calculated with more than ten inoculation sites for each individual plant. The gel image indicates the plants carrying pU1301::OsPGIP1 by PCR amplification with the primer pair of Hpt-F/R. *Bars* represent the means ± SD. Significant differences were determined by *t* test: *P < 0.05 and **P < 0.01, respectively.

**Additional file 9: Figure S3.** Three *OsPGIP1*-silenced lines enhanced the susceptibility of ZH11 to bacterial leaf streak in the T_1_ generation. Cosegregation of the lesion length with PCR positive selection for three *OsPGIP1*-silenced rice lines, RNAi-7, RNAi-10 and RNAi-11, in the ZH11 background. The average lesion length was measured with over ten inoculation sites for each individual plant. The gel image indicates the plants carrying ds1301::OsPGIP1 by PCR amplification with the primer pair Hpt-F/R. Bars represent the means ± SD. Significant differences were determined by *t* test: *P < 0.05 and **P < 0.01, respectively.

**Additional file 10: Figure S4.** Repressing the *OsPGIP1* expression enhanced the susceptibility of Acc8558 to BLS in the T_1_ generation. Cosegregation of the lesion length with PCR positive selection for three *OsPGIP1* RNAi lines, RNAi-1, RNAi-4 and RNAi-7 in moderately resistant rice variety Acc8558 background. The average lesion length was calculated with over ten inoculation sites for each individual plant. The gel image indicates the plants carrying ds1301::OsPGIP1 by PCR amplification with the primer pair Hpt-F/R. *Bars* represent the means ± SD. Significant differences were determined by *t* test: *P < 0.05 and **P < 0.01, respectively.

**Additional file 11: Figure S5.** Bacterial growth curve of RS105 in *OsPGIP1*-overexpressing and *OsPGIP1*-silenced rice lines. The bacterial populations of RS105 in the wild-type ZH11 rice, *OsPGIP1*-overexpressing rice line OV-24 and *OsPGIP1*-silenced rice line RNAi-10 were detected at 1, 4 and 7 days postinoculation. Significant differences were determined by *t* test: *P < 0.05.

**Additional file 12: Figure S6.** XocPG fails to interact with OsPGIP1 or OsPGIP4 by yeast two hybridization. The yeast transformants grew on double-dropout minimal base (SD/−leucine-tryptophan) and the interaction in yeast was tested by quadruple-dropout minimal base (SD/−leu-trp-ade-his) and aureobasidin A. The pGBKT7–53 and pGADT7-T yeast transformation was used as the positive control.

**Additional file 13: Figure S7.** GO analysis of the DEGs in *OsPGIP1*-overexpressing rice. (a) The functional analysis of genes that were upregulated and downregulated in OV-24 compared with ZH11 without RS105 inoculation. (b) The Venn diagram of DEGs in OV-24 compared with ZH11 (ZH11vs OV-24), ZH11 inoculated with RS105 compared to ZH11 (ZH11 vs ZH11-RS), and OV-24 inoculated with RS105 compared to OV-24 (OV-24 vs OV-24-RS).

**Additional file 14: Figure S8.** GO analysis of DEGs uniquely regulated in the *OsPGIP1*-overexpressing rice. The GO analysis of DEGs that specifically changed in *OsPGIP1* OV-24-RS included cellular component, molecular function and biological process categories.

**Additional file 15: Figure S9.** The yield traits of *OsPGIP1*-overexpressing rice showed no significant changes. (a) The tiller number of the two *OsPGIP1* OV lines (OV-12 and OV-24) and ZH11 were counted in at least 30 individual plants after the full growth period. (b) The OV-12, OV-24 and ZH11 rice seeds were harvested after the complete growth period and after removing moisture with a dryer. Then 1000 seed grains were weighed for the rice, and the experiment was repeated 10 times.

## Data Availability

The data sets supporting the results of this article are included within the article and its additional files. The RNA-seq data supporting the results of this article are available in the NCBI’s SRA with the accession number PRJNA517024 (http://trace.ncbi.nlm.nih.gov/Traces/sra). The accession numbers of related genes in this research are listed in the Additional file 2: Table S1.These genes can be searched out in NCBI (http://www.ncbi.nlm.nih.gov/) and Rice Genome Annotation Project (http://www.rice.plantbiology.msu.edu/).

## Abbreviations

BLS: Bacterial leaf streak
CWDEs: Cell wall-degrading enzymes
DAMP: Damage-associated molecular pattern
DEG: Differentially expressed gene
dpi: Days post inoculation
DR: Defense-related
hpi: Hours postinoculation
OGs: Oligogalacturonides
OV: Overexpressing
PG: Polygalacturonase
PGIP: Polygalacturonase-inhibiting protein
PR: Pathogenesis related
SB: Sheath blight
WAK1: Wall-associated kinase 1
WT: wild-type
*Xoc*: *Xanthomons oryzae* pv. *oryzicola*
