# Supplementary material for: OsPGIP1-Mediated Resistance to Bacterial Leaf Streak in Rice is Beyond Responsive to the Polygalacturonase of Xanthomonas oryzae pv. oryzicola
Source: Rice (N Y). 2019 Dec 12;12:90. doi: 10.1186/s12284-019-0352-4 (PMC6908543; doi:10.1186/s12284-019-0352-4)
Supplement: Supplementary file 1 — Additional file 1: Text S1. Comparison of the OsPGIP1sequence containing CDS and promoter between ZH11 and Acc8558. [file 12284_2019_352_MOESM1_ESM.docx]

**Text S1.** Comparison of the *OsPGIP1*sequence containing CDS and promoter between ZH11 and Acc8558.

ZH11 1 -ACACGGGAGAAAAAATCGTAATTAGTTTACTACTCCCTCCGTCCTAGAATATAAGAGATTTTAAGTTTTTTCTTGCAACGTTTGACCACTCGTCTTATT 99

.|......|.|.||||||||||||||||||||||||||||||||.||||||||||.||||||.|||||||||||||||||||.||||||||||||||||

Acc8558 1 TTCCGACCGGGAGAAATCGTAATTAGTTTACTACTCCCTCCGTCCCAGAATATAAGGGATTTTGAGTTTTTTCTTGCAACGTTCGACCACTCGTCTTATT 100

ZH11 100 CAATTTTTTTAAAAATTATTATTTATTTTATTTGTGACTTACTTTATTATCCACAGTATTTTAAGCACAACTTTTCATTTTTTATATTTGCAAAAAAAAT 199

||||||||||.|||||||||||||||||||||||||||||||||||||||||||||||||||||||||||||||||||||||||||||||||||||||||

Acc8558 101 CAATTTTTTTTAAAATTATTATTTATTTTATTTGTGACTTACTTTATTATCCACAGTATTTTAAGCACAACTTTTCATTTTTTATATTTGCAAAAAAAAT 200

ZH11 200 TTGAATAATACGAGTGGTCAAATGTTGCAAGCAAAAACTCAAAACCCCTTATATTGTGGGACGGAGGGAGTAATGATTAGTCTCCTATTTTTACTACTGA 299

|||||||||||||||||||||||||||||||.||||||||||||||||||||||||||||||||||||||||||||||||||||||||||||||||||||

Acc8558 201 TTGAATAATACGAGTGGTCAAATGTTGCAAGTAAAAACTCAAAACCCCTTATATTGTGGGACGGAGGGAGTAATGATTAGTCTCCTATTTTTACTACTGA 300

ZH11 300 TTAGTTTACTAGTAATTTTTAAATCTTAACTTAAAAATTTTCAAATTTGGACTTGAAAGTTTTCAAATCTCGAGCTAAAAATTTTCAAATCTGGACTTGA 399

||||||||||||||||||||||||||||||||||||||||||||||||||||||||||||||||||||||||||.||||||

Acc8558 301 TTAGTTTACTAGTAATTTTTAAATCTTAACTTAAAAATTTTCAAATTTGGACTTGAAAGTTTTCAAATCTCGAGTTAAAAA------------------- 381

ZH11 400 AAGTTTTTAAATCTGAGCTCAAAGTTTTCAAATCTCGAGTTGAAAGTTTTCAAAATTTTCAAATCTGGACTTGAAAGTTTTTAAATCTGAGCTCAAAGTT 499

|||||||||||.|||||.||||||||||||||||||||||||||||||||||||||||||||||||||||||||||

Acc8558 382 ------------------------TTTTCAAATCTTGAGTTAAAAGTTTTCAAAATTTTCAAATCTGGACTTGAAAGTTTTTAAATCTGAGCTCAAAGTT 457

ZH11 500 TTCAAATCTCGAGTTGAAAGTTTTCAAAATTTTCAAATCTGGACTTGAAAATTTTCGAATCTCGAGTTGAAAGTTTTTCAAATGTGAGATGAAAAGTTAA 599

||||||||||||||||||||||||||||||||||||||||||||||||||||||||||||||||||||||||| ||||||||||||||||||||||||||

Acc8558 458 TTCAAATCTCGAGTTGAAAGTTTTCAAAATTTTCAAATCTGGACTTGAAAATTTTCGAATCTCGAGTTGAAAG-TTTTCAAATGTGAGATGAAAAGTTAA 556

ZH11 600 AAGTTTTTAAAATTTGACTTAAAAAAAAATTCAAATTTCGAGTTAAAATTTTCAAATCTGGATTTAAAATTTTGAAAATTTGACTTCAGAAATAGATTTC 699

|||||||||||||||||||| |||||||||||||||||||||||||||.|||||||||||||||||||||||||||||||||||||||||||||||

Acc8558 557 AAGTTTTTAAAATTTGACTT-----AAAATTCAAATTTCGAGTTAAAATTTTTAAATCTGGATTTAAAATTTTGAAAATTTGACTTCAGAAATAGATTTC 651

ZH11 700 TTTTCAATTTGTTAGTTAAAAAATCTTGTGAAAAAAAGAAAATCCTAACTACTACTGTCATTATCTTAAGCTATAATTATCTAAAACTAATTAATTGATT 799

||||||||||||||||||||||||||||||||||||||||||||||||||||||||||||||||||||||||||||||||||||||||||||||||||||

Acc8558 652 TTTTCAATTTGTTAGTTAAAAAATCTTGTGAAAAAAAGAAAATCCTAACTACTACTGTCATTATCTTAAGCTATAATTATCTAAAACTAATTAATTGATT 751

ZH11 800 AGTTAACTATACCTCCTATCTTTAGTCCCGAAACGCGCGTGGGGGGCGCGCACGCCCCAACTAG--GCCCCCCCCAATGCCTGAATCGCACTAAACATGC 897

||||||||||||||||||.||||||||||||||||||.|.||||||.||||||||||||||||| .||||||||||||||.||||||||||||||||||

Acc8558 752 AGTTAACTATACCTCCTACCTTTAGTCCCGAAACGCGTGGGGGGGGGGCGCACGCCCCAACTAGCCCCCCCCCCCAATGCCCGAATCGCACTAAACATGC 851

ZH11 898 GTGTGCTTATGTAAAGTTAACAAACTAAGGGGAAAAAAAATCAAAGTGTCTTGTAATATGAAACGGGATAGTATAAATTTTAGATAATGGGAAAATGATC 997

|.|||||||||||||||||||||||||||||| |||||||||||||.|.|||||||||||||||||||||||||||||||||.|||||||||||||||||

Acc8558 852 GGGTGCTTATGTAAAGTTAACAAACTAAGGGG-AAAAAAATCAAAGGGGCTTGTAATATGAAACGGGATAGTATAAATTTTAAATAATGGGAAAATGATC 950

ZH11 998 ATTCTGTCATTTGTACCATACGCATCATCTCCACCATAGATACTCCCTTTGTCTAGAAATATGAGCATTTATAGAAGTCTTATACTCTGACTATCTTAAA 1097

||||||||||||||||||||||||||||||||.|||||.|.||||||||||||||.|||||||||||||||||.|||||||||||||.||||||||||||

Acc8558 951 ATTCTGTCATTTGTACCATACGCATCATCTCCCCCATAAAAACTCCCTTTGTCTAAAAATATGAGCATTTATAAAAGTCTTATACTCCGACTATCTTAAA 1050

ZH11 1098 AGATTATATTTTTTGAAGCTAGCTAATATCTGACAGATTAGTTCAGATGTCAAAGGATGATCATGCCTTTCATTAAGACTGTTTTCTATGCTTCGACGCT 1197

|||||||||||||||||||||||||||||||||||||||||||||||||||||||||||||||||||||||||||||||||||||||||||||.||||||

Acc8558 1051 AGATTATATTTTTTGAAGCTAGCTAATATCTGACAGATTAGTTCAGATGTCAAAGGATGATCATGCCTTTCATTAAGACTGTTTTCTATGCTTGGACGCT 1150

ZH11 1198 CGGCTAAACTTTTGGCATAGCCAAGCTCTAATACGGGACATTTGCAACTGACAATTGGAGGCATGGTCTCTACTTAAGCATAGAAGAGAAACGAAGATGA 1297

|||||||||||||||||||||||| |||||||||||||||||||||||||||||||||||||||||||||||||||||||||||||||||

Acc8558 1151 CGGCTAAACTTTTGGCATAGCCAA-----------GGACATTTGCAACTGACAATTGGAGGCATGGTCTCTACTTAAGCATAGAAGAGAAACGAAGATGA 1239

ZH11 1298 TAAAGCTTAATTTTGTGGATGGAGCGAGTAGATGCACAAGATAAGAATAGAAAACTGGATATTTGTGGGACAAG-------------------------- 1371

||||||||||||||||||||||||||||||||||||||||||||||||||||||.|||||||||||||||||||

Acc8558 1240 TAAAGCTTAATTTTGTGGATGGAGCGAGTAGATGCACAAGATAAGAATAGAAAATTGGATATTTGTGGGACAAGAAGATCAATAATGTTCCCATGGATAG 1339

ZH1 1372 -------------AAGATCAATAAGCAAGCAGAAGAATATTATTTGTCCTAGATCAATAAGGTGCTCTGTCCCTGCACAGGCGTGGTAGGCCTGGGTTGA 1458

||||||||||||||||||||||||||||||||||||||||||||||||||||||||||||||||||||||||||||||||||||||

Acc8558 1340 ATGATGGTGGTCCAAGATCAATAAGCAAGCAGAAGAATATTATTTGTCCTAGATCAATAAGGTGCTCTGTCCCTGCACAGGCGTGGTAGGCCTGGGTTGA 1439

ZH11 1459 CTCTCCATTTGGACAAGTTTGGCAATTTGGCACTGCCAACTTTGACCATTTCATTCTCCCTGGCTTTCCCGTGCTTGCTACAGTATTTGTATTTTATCTC 1558

||||||||||||||||||||||||||||||||||||||||||||||||||||||||||||||||||||||||||||||||||||||||||||||||||||

Acc8558 1440 CTCTCCATTTGGACAAGTTTGGCAATTTGGCACTGCCAACTTTGACCATTTCATTCTCCCTGGCTTTCCCGTGCTTGCTACAGTATTTGTATTTTATCTC 1539

ZH11 1559 CTACTCCTACTTTGGCTTGGCTGGCTCCCTGGCGGCTTCAAGAAACTGCGTCTGCGGCGCCTCACACTCACATGCATGTGCATTAAATGAGCAGAGCTGT 1658

||||||||||||||||||||||||||||||||||||||||||||||||||||||||||||||||||||||||||||||||||||||||||||||||||||

Acc8558 1540 CTACTCCTACTTTGGCTTGGCTGGCTCCCTGGCGGCTTCAAGAAACTGCGTCTGCGGCGCCTCACACTCACATGCATGTGCATTAAATGAGCAGAGCTGT 1639

ZH11 1659 ACCCTTCTCATCCGCATGCACGCTTGCATGGGTACGGTGGTCAATGTTTTTTGTTGACTCGCTATTGCATGCGTTGGATTTTTTTTTCAACTTGATTCTT 1758

|||||||||||||||||||||||| |||||||||||||||||||||||||||||||||||||||||||| |||||||||||||||||||||

Acc8558 1640 ACCCTTCTCATCCGCATGCACGCT----------CGGTGGTCAATGTTTTTTGTTGACTCGCTATTGCATGCGTTGGA-TTTTTTTTCAACTTGATTCTT 1728

ZH11 1759 CTTCCTTGCGATTAATTTTCCAAATAATTTCATACAACAAGTCAACAACAACAGTTGATTAATACCAACCATCAAATTAATCTCCCTCTCCCCCTTTCTC 1858

||||||||||||||||||||||||||||||||||||||||||||||||||||||||||||||||||||||||||||||||||||||||||||||||||||

Acc8558 1729 CTTCCTTGCGATTAATTTTCCAAATAATTTCATACAACAAGTCAACAACAACAGTTGATTAATACCAACCATCAAATTAATCTCCCTCTCCCCCTTTCTC 1828

ZH11 1859 TCTATATAACACATCTCATCATCTAGCTCACTCACTGAAATGCCCAACTAAATTGGAGTGGAGGCTACTACCTAATTAATACGATATCCTACAAGAACAA 1958

||||||||||||||||||||||||||||||||||||||||||||||||||||||||||||||||||||||||||||||||||||||||||||||||||||

Acc8558 1829 TCTATATAACACATCTCATCATCTAGCTCACTCACTGAAATGCCCAACTAAATTGGAGTGGAGGCTACTACCTAATTAATACGATATCCTACAAGAACAA 1928

ZH11 1959 GAATGCGCGCCATGGTCGTCGTTCTTGTGCTCGCCGCGGCCGGCGCCGCCGCTGCCACCACGAAGAAGAAGGAGTGCAACGCCGGCGACAAGGCCGCACT 2058

||||||||||||||||||||||||||||||||||||||||||||||||||||||||||||||||||||||||||||||||||||||||||||||||||||

Acc8558 1929 GAATGCGCGCCATGGTCGTCGTTCTTGTGCTCGCCGCGGCCGGCGCCGCCGCTGCCACCACGAAGAAGAAGGAGTGCAACGCCGGCGACAAGGCCGCACT 2028

ZH11 2059 GCTGGCCATCAAGAAGGCCCTCGGCGACCCCTACCACTTCGCCTCCTGGACGCCCGACAACCTCTGCTGCGAATGGTACGACGTCACCTGCGACGACACC 2158

||||||||||||||||||||||||||||||||||||||||||||||||||||||||||||||||||||||||||||||||||||||||||||||||||||

Acc8558 2029 GCTGGCCATCAAGAAGGCCCTCGGCGACCCCTACCACTTCGCCTCCTGGACGCCCGACAACCTCTGCTGCGAATGGTACGACGTCACCTGCGACGACACC 2128

ZH11 2159 ACCGACCGCGTCGTCGGCCTCTCCGTCTTCCAGGACGCCAACCTCACCGGCACCATCCCCGACGCCGTCGCCGGCCTCACCCACCTCCGCACCCTCACGT 2258

||||||||||||||||||||||||||||||||||||||||||||||||||||||||||||||||||||||||||||||||||||||||||||||||||||

Acc8558 2129 ACCGACCGCGTCGTCGGCCTCTCCGTCTTCCAGGACGCCAACCTCACCGGCACCATCCCCGACGCCGTCGCCGGCCTCACCCACCTCCGCACCCTCACGT 2228

ZH11 2259 GGCACCACCTCCCCCAGATCTCCGGCCCCATCCCGCCGGCGATCGCCAAGCTCAACCGCCTCTCCCTCCTCATCATCTCCTGGACCGCCGTCTCCGGCCC 2358

||||||||||||||||||||||||||||||||||||||||||||||||||||||||||||||||||||||||||||||||||||||||||||||||||||

Acc8558 2229 GGCACCACCTCCCCCAGATCTCCGGCCCCATCCCGCCGGCGATCGCCAAGCTCAACCGCCTCTCCCTCCTCATCATCTCCTGGACCGCCGTCTCCGGCCC 2328

ZH11 2359 CGTCCCCTCCTTCCTCGGCGGGCTCAAGAGCCTCACCCTCCTCGATCTCTCCTTCAACTCCCTCACCGGCGCCATCCCGCCGTCGCTCGCCGCCCTCCCC 2458

||||||||||||||||||||||||||||||||||||||||||||||||||||||||||||||||||||||||||||||||||||||||||||||||||||

Acc8558 2329 CGTCCCCTCCTTCCTCGGCGGGCTCAAGAGCCTCACCCTCCTCGATCTCTCCTTCAACTCCCTCACCGGCGCCATCCCGCCGTCGCTCGCCGCCCTCCCC 2428

ZH11 2459 TTCCTCAGCGGCATCGACATCAGCCGCAACCGCCTCACCGGCCCCCTGCCGCCGGCGCTCTTCAGCAAGCTCAACACCACGCAGCAGGGCGGCGCCTACC 2558

||||||||||||||||||||||||||||||||||||||||||||||||||||||||||||||||||||||||||||||||||||||||||||||||||||

Acc8558 2429 TTCCTCAGCGGCATCGACATCAGCCGCAACCGCCTCACCGGCCCCCTGCCGCCGGCGCTCTTCAGCAAGCTCAACACCACGCAGCAGGGCGGCGCCTACC 2528

ZH11 2559 TGAGGCTGTCGCGGAACAACCTCACCGGCGGCATCCCGGCCGAGTACGGCGGCGTGGCGTTCGAGGTGATGGACCTGTCGCGCAACGCGCTGAGCTTCGA 2658

||||||||||||||||||||||||||||||||||||||||||||||||||||||||||||||||||||||||||||||||||||||||||||||||||||

Acc8558 2529 TGAGGCTGTCGCGGAACAACCTCACCGGCGGCATCCCGGCCGAGTACGGCGGCGTGGCGTTCGAGGTGATGGACCTGTCGCGCAACGCGCTGAGCTTCGA 2628

ZH11 2659 CATGACCGGGCTGCGGCTGCAGGAAGGGGTGAGCTCGCTGGACCTCAGCCACAACATGCTGTACGGCGGCGTGCCGGCGCAGGTGGCCGGGCTGAGCAGC 2758

||||||||||||||||||||||||||||||||||||||||||||||||||||||||||||||||||||||||||||||||||||||||||||||||||||

Acc8558 2629 CATGACCGGGCTGCGGCTGCAGGAAGGGGTGAGCTCGCTGGACCTCAGCCACAACATGCTGTACGGCGGCGTGCCGGCGCAGGTGGCCGGGCTGAGCAGC 2728

ZH11 2759 CTGCAGGACTTCAACGTCAGCTACAACCGCCTCTGCGGCGAGCTGCCCGCCGGCGCCGCGAGGTTCGATCAGTACTCCTTCTTCCACAACAAGTGCCTCT 2858

||||||||||||||||||||||||||||||||||||||||||||||||||||||||||||||||||||||||||||||||||||||||||||||||||||

Acc8558 2729 CTGCAGGACTTCAACGTCAGCTACAACCGCCTCTGCGGCGAGCTGCCCGCCGGCGCCGCGAGGTTCGATCAGTACTCCTTCTTCCACAACAAGTGCCTCT 2828

ZH11 2859 GCGGACCGCCGCTCCCTACTCCCTGCAATTAAGCTCCCATTACTTAATTACTAGCTATGTGGTGAAATTATACATCAGCAATGCAAGCAAAATAAATAAA 2958

||||||||||||||||||||||||||||||||||||||||||||||||||||||||||||||||||||||||||||||||||||||||||||||||||||

Acc8558 2829 GCGGACCGCCGCTCCCTACTCCCTGCAATTAAGCTCCCATTACTTAATTACTAGCTATGTGGTGAAATTATACATCAGCAATGCAAGCAAAATAAATAAA 2928

ZH11 2959 TAAATATATATATATATATATATATATATATATATATATATATATATATATATATATATATATTTGTATAATGATTTTAATAAGTCAAAAACAGATTGAT 3058

||||||||||||||||||||||||||||||||||||||||||||||||||||||||||||||||||||||||||||||||||||||||||||||||||||

Acc8558 2929 TAAATATATATATATATATATATATATATATATATATATATATATATATATATATATATATATTTGTATAATGATTTTAATAAGTCAAAAACAGATTGAT 3028

ZH11 3059 GCATTTGGAAGGAATGATTAATAAAGCTGTGTTCAATTGTTTCTGGGACAATTAA 3113

|||||||||||||||||||||||||||||||||||||||||||||||||||||||

Acc8558 3029 GCATTTGGAAGGAATGATTAATAAAGCTGTGTTCAATTGTTTCTGGGACAATTAA 3083
