# Supplementary figures and images for: OsPGIP1-Mediated Resistance to Bacterial Leaf Streak in Rice is Beyond Responsive to the Polygalacturonase of Xanthomonas oryzae pv. oryzicola
Source: Rice (N Y). 2019 Dec 12;12:90. doi: 10.1186/s12284-019-0352-4 (PMC6908543; doi:10.1186/s12284-019-0352-4)

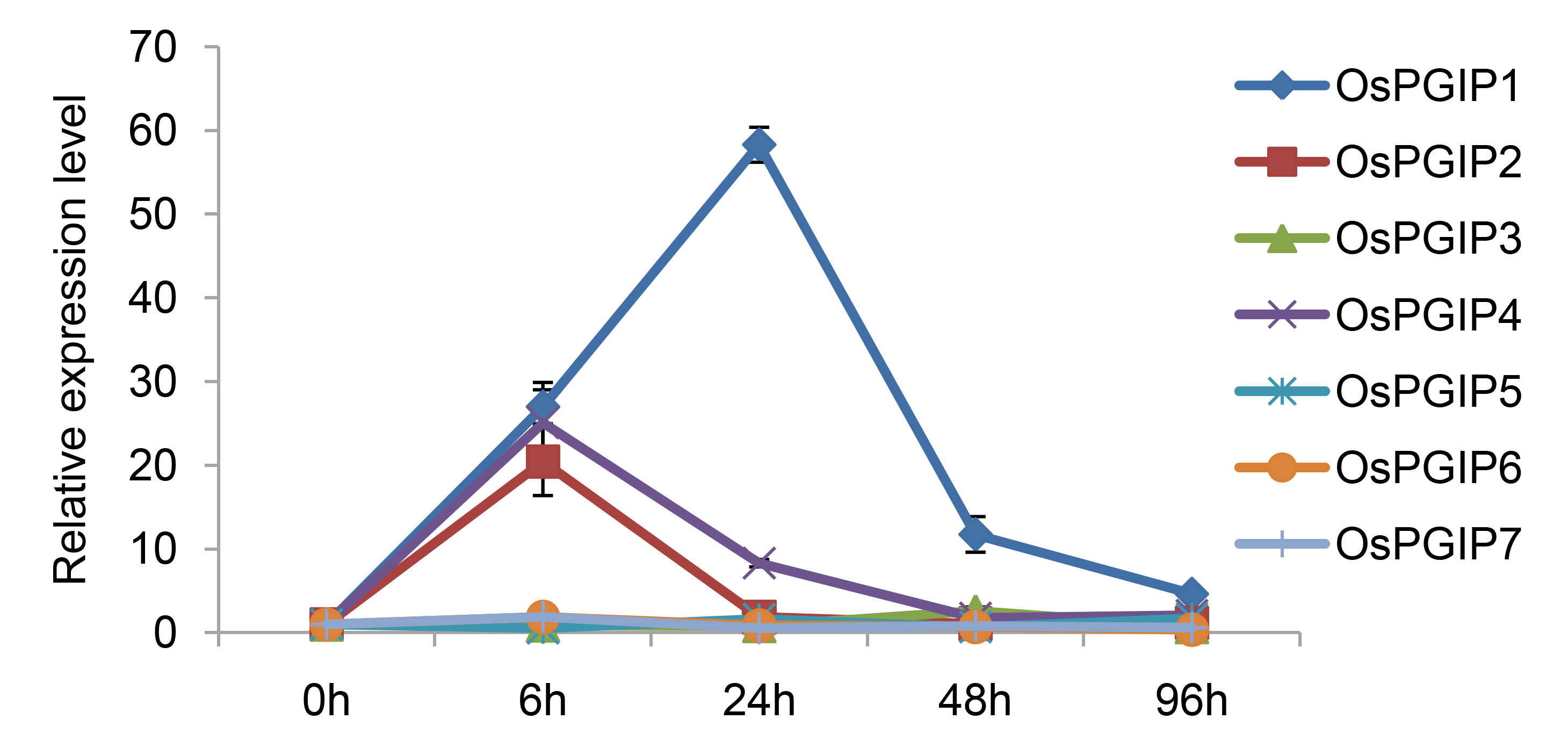

Supplement: Supplementary file 7 — Additional file 7: Figure S1. Expression patterns of rice OsPGIPs in response to RS105 in Acc8558 rice. The expression of OsPGIPs in BLS moderately resistant rice variety Acc8558 at 6, 24, 48, and 96 h postinoculation was related to leaves without inoculation (0 h). The ACTIN was used as an internal control. Error bars represent the standard deviations for three replicates. [file 12284_2019_352_MOESM7_ESM.jpg]

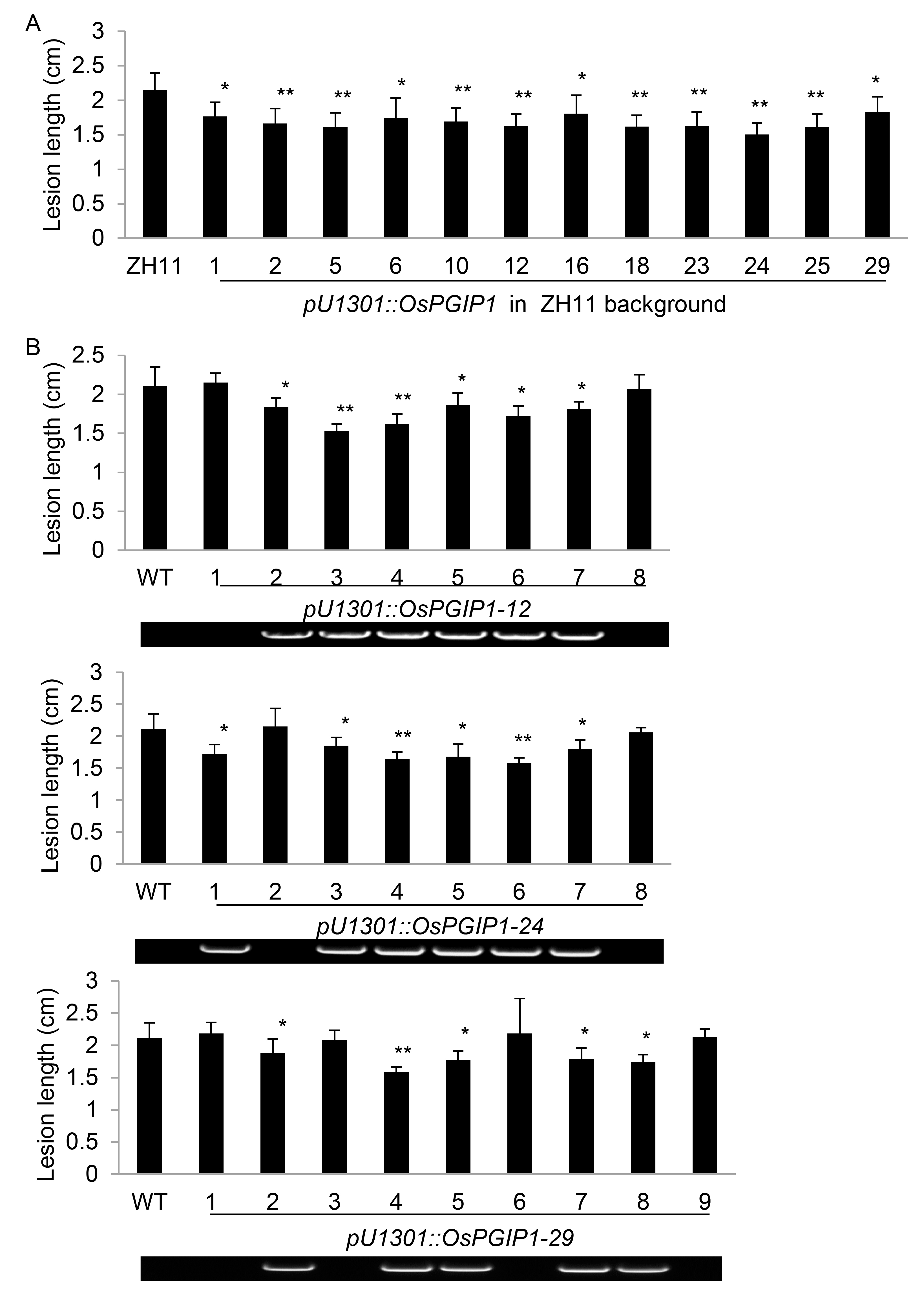

Supplement: Supplementary file 8 — Additional file 8: Figure S2. Resistance of the OsPGIP1-overexpressing plants to the Xoc strain RS105 in the T0 and T1 generation. (a) Lesion length analysis of OsPGIP1-overexpressing transgenic rice in the T0 generation in a ZH11 background 14 days after inoculation with RS105. (b) Cosegregation of the lesion length with PCR positive selection in the T1 generation for the OV-12, OV-24 and OV-29 lines. The average lesion length was calculated with more than ten inoculation sites for each individual plant. The gel image indicates the plants carrying pU1301::OsPGIP1 by PCR amplification with the primer pair of Hpt-F/R. Bars represent the means ± SD. Significant differences were determined by t test: *P < 0.05 and **P < 0.01, respectively. [file 12284_2019_352_MOESM8_ESM.jpg]

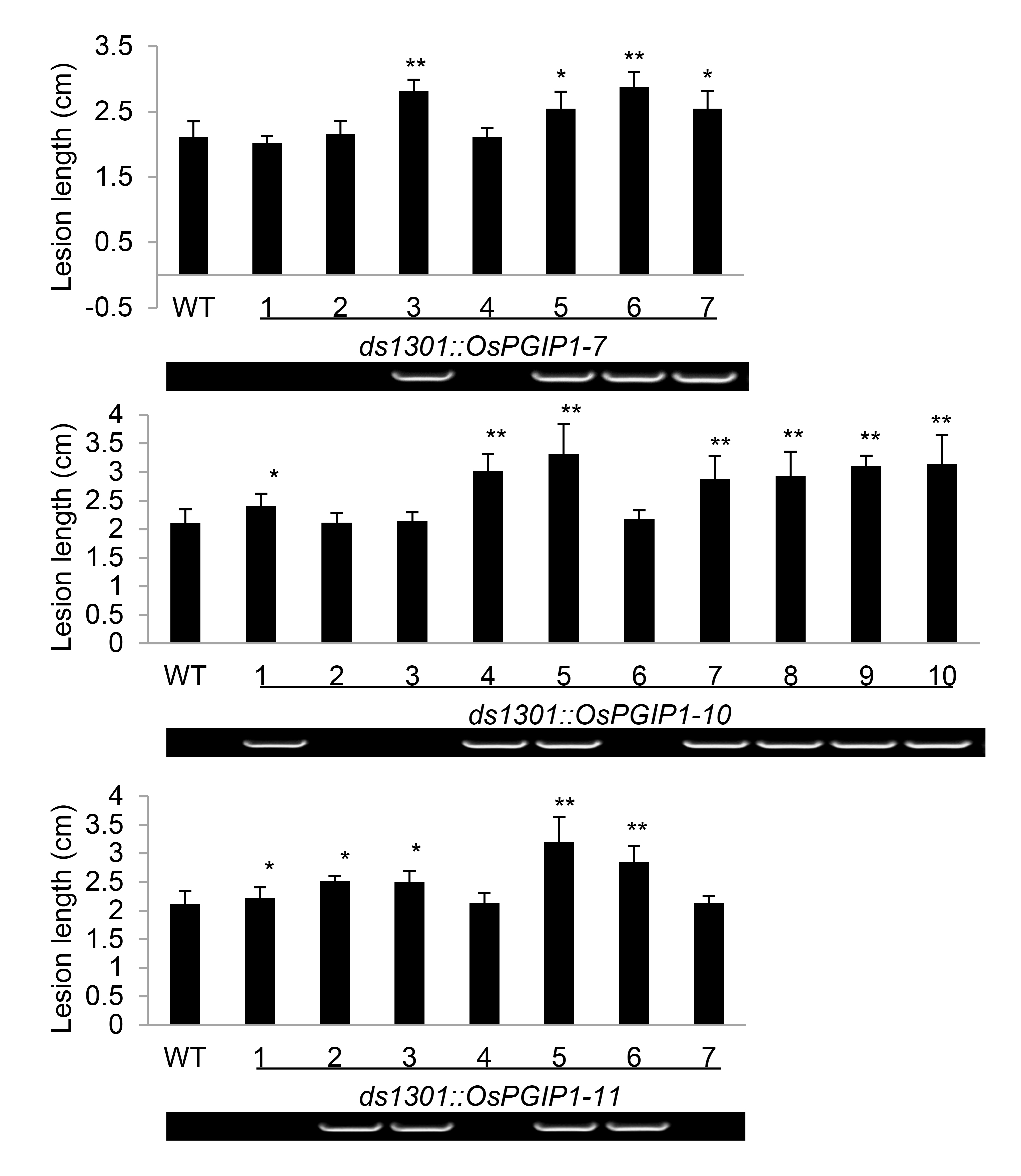

Supplement: Supplementary file 9 — Additional file 9: Figure S3. Three OsPGIP1-silenced lines enhanced the susceptibility of ZH11 to bacterial leaf streak in the T1 generation. Cosegregation of the lesion length with PCR positive selection for three OsPGIP1-silenced rice lines, RNAi-7, RNAi-10 and RNAi-11, in the ZH11 background. The average lesion length was measured with over ten inoculation sites for each individual plant. The gel image indicates the plants carrying ds1301::OsPGIP1 by PCR amplification with the primer pair Hpt-F/R. Bars represent the means ± SD. Significant differences were determined by t test: *P < 0.05 and **P < 0.01, respectively. [file 12284_2019_352_MOESM9_ESM.jpg]

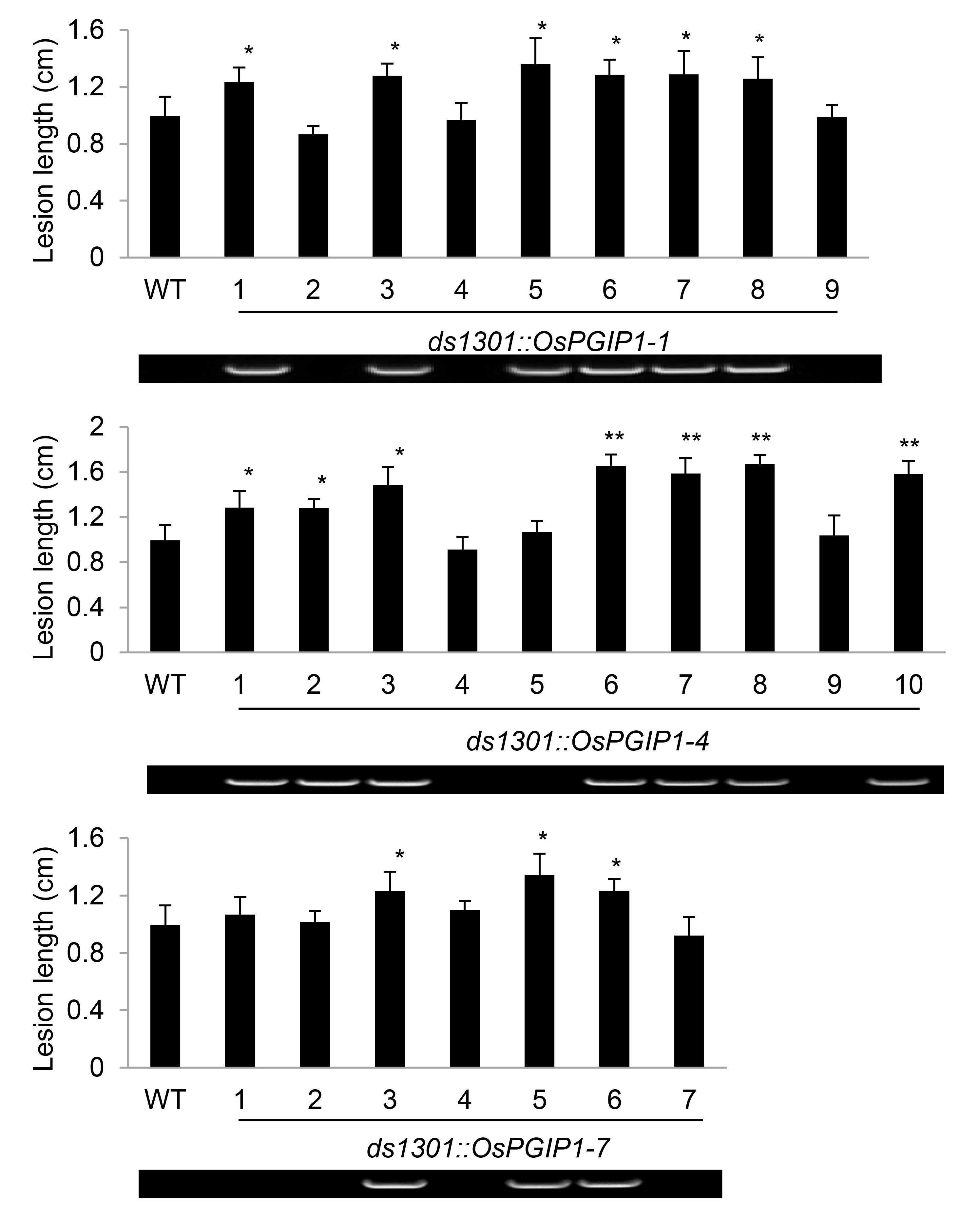

Supplement: Supplementary file 10 — Additional file 10: Figure S4. Repressing the OsPGIP1 expression enhanced the susceptibility of Acc8558 to BLS in the T1 generation. Cosegregation of the lesion length with PCR positive selection for three OsPGIP1 RNAi lines, RNAi-1, RNAi-4 and RNAi-7 in moderately resistant rice variety Acc8558 background. The average lesion length was calculated with over ten inoculation sites for each individual plant. The gel image indicates the plants carrying ds1301::OsPGIP1 by PCR amplification with the primer pair Hpt-F/R. Bars represent the means ± SD. Significant differences were determined by t test: *P < 0.05 and **P < 0.01, respectively. [file 12284_2019_352_MOESM10_ESM.jpg]

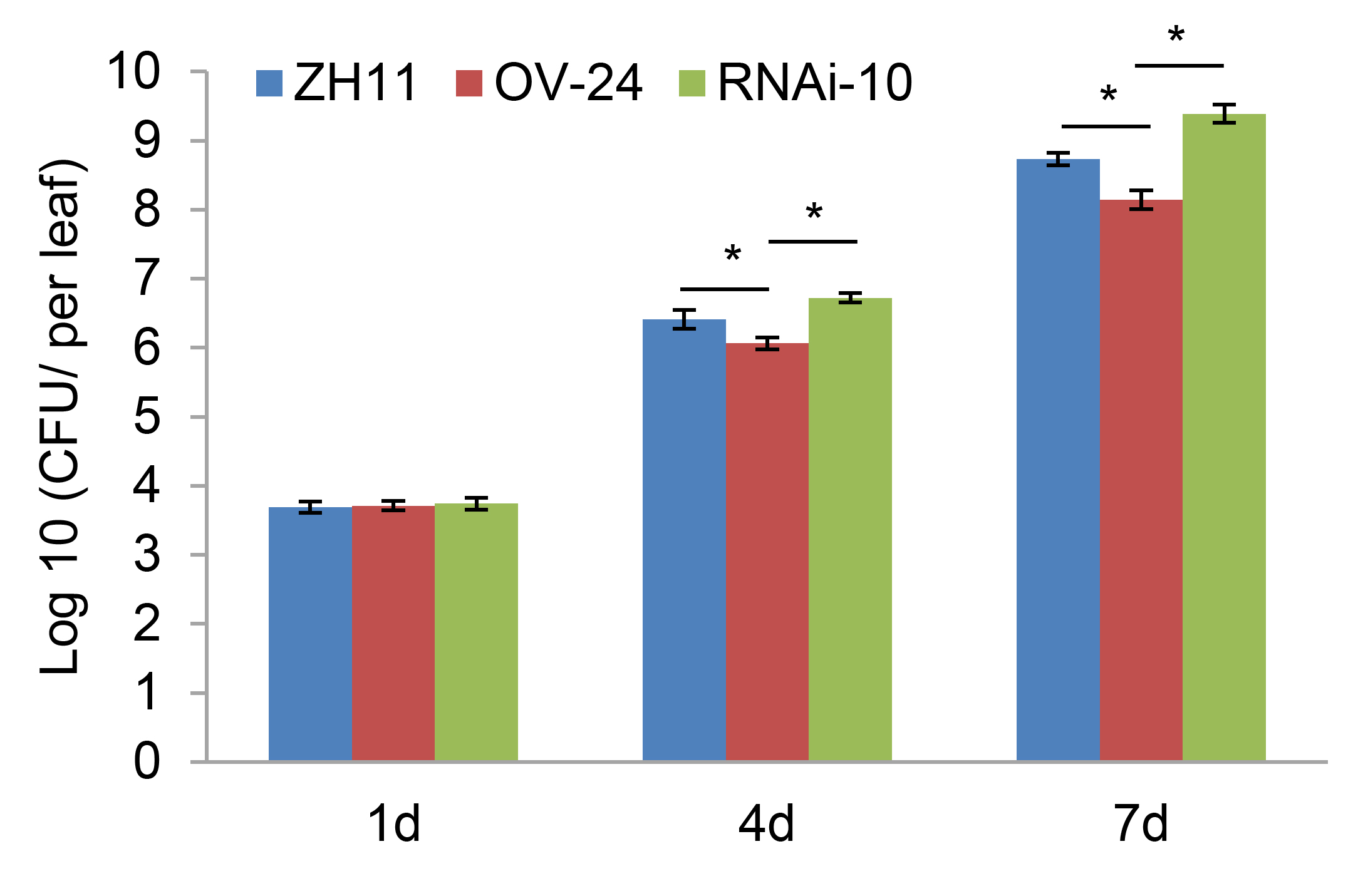

Supplement: Supplementary file 11 — Additional file 11: Figure S5. Bacterial growth curve of RS105 in OsPGIP1-overexpressing and OsPGIP1-silenced rice lines. The bacterial populations of RS105 in the wild-type ZH11 rice, OsPGIP1-overexpressing rice line OV-24 and OsPGIP1-silenced rice line RNAi-10 were detected at 1, 4 and 7 days postinoculation. Significant differences were determined by t test: *P < 0.05. [file 12284_2019_352_MOESM11_ESM.jpg]

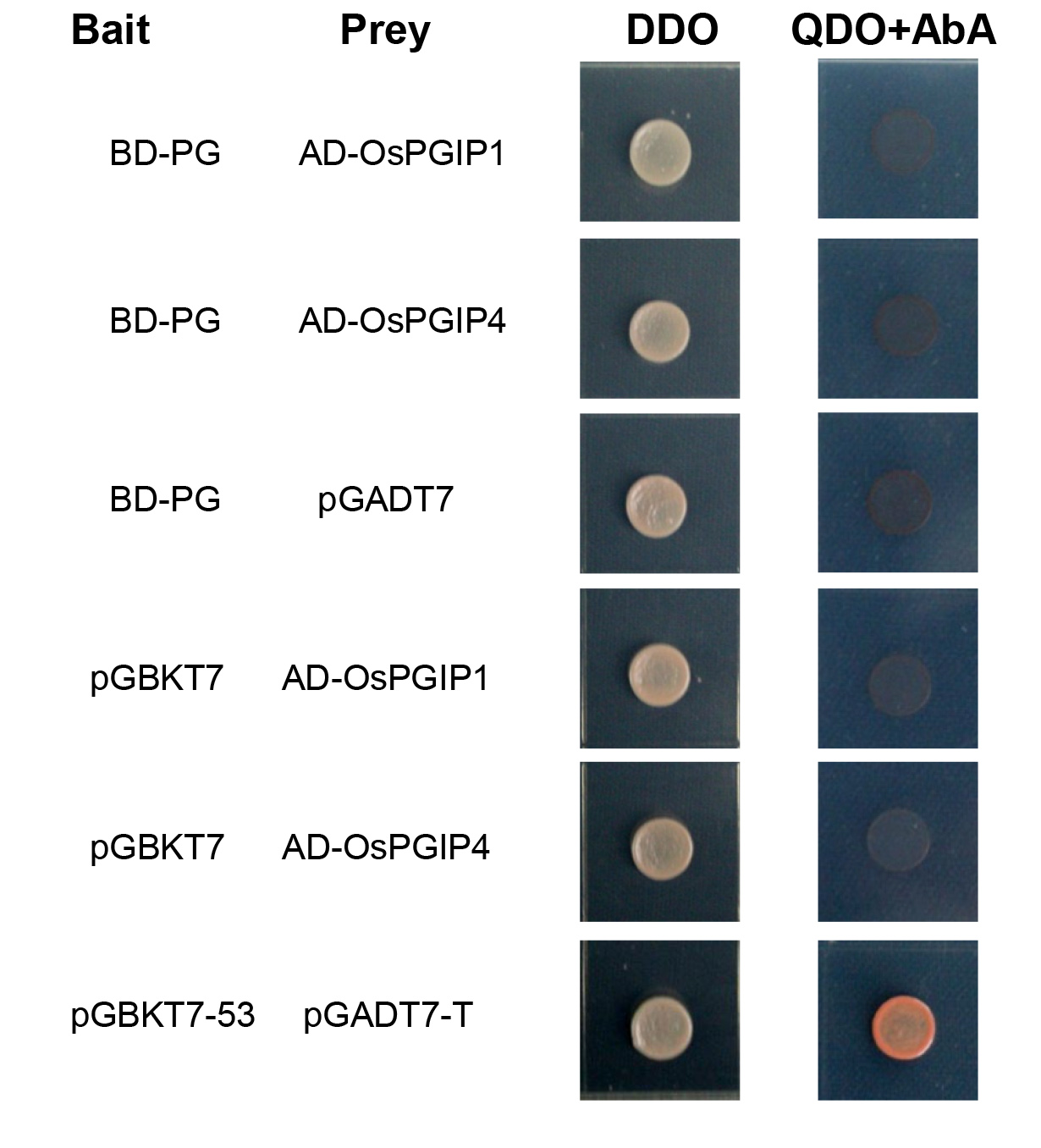

Supplement: Supplementary file 12 — Additional file 12: Figure S6. XocPG fails to interact with OsPGIP1 or OsPGIP4 by yeast two hybridization. The yeast transformants grew on double-dropout minimal base (SD/−leucine-tryptophan) and the interaction in yeast was tested by quadruple-dropout minimal base (SD/−leu-trp-ade-his) and aureobasidin A. The pGBKT7–53 and pGADT7-T yeast transformation was used as the positive control. [file 12284_2019_352_MOESM12_ESM.jpg]

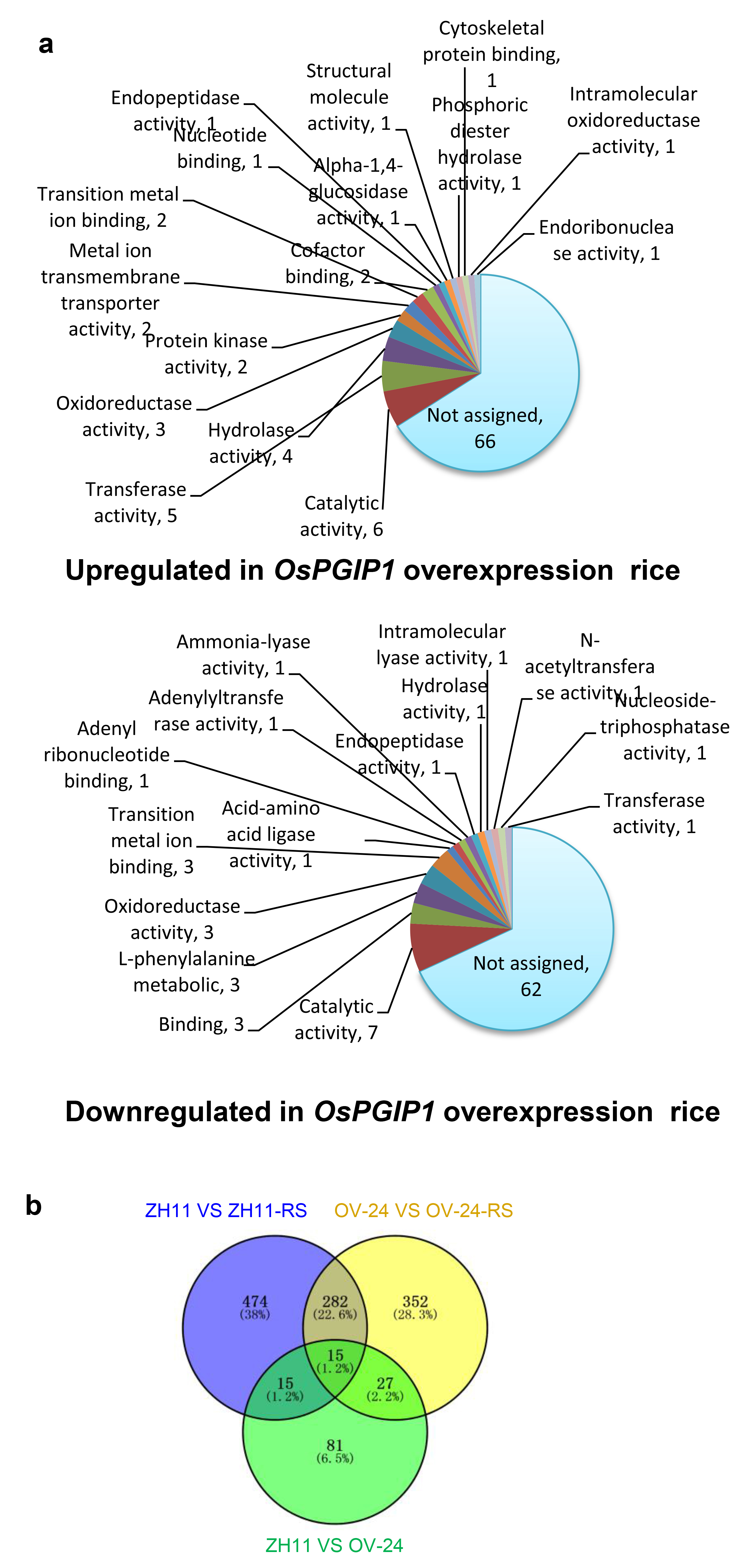

Supplement: Supplementary file 13 — Additional file 13: Figure S7. GO analysis of the DEGs in OsPGIP1-overexpressing rice. (a) The functional analysis of genes that were upregulated and downregulated in OV-24 compared with ZH11 without RS105 inoculation. (b) The Venn diagram of DEGs in OV-24 compared with ZH11 (ZH11vs OV-24), ZH11 inoculated with RS105 compared to ZH11 (ZH11 vs ZH11-RS), and OV-24 inoculated with RS105 compared to OV-24 (OV-24 vs OV-24-RS). [file 12284_2019_352_MOESM13_ESM.jpg]

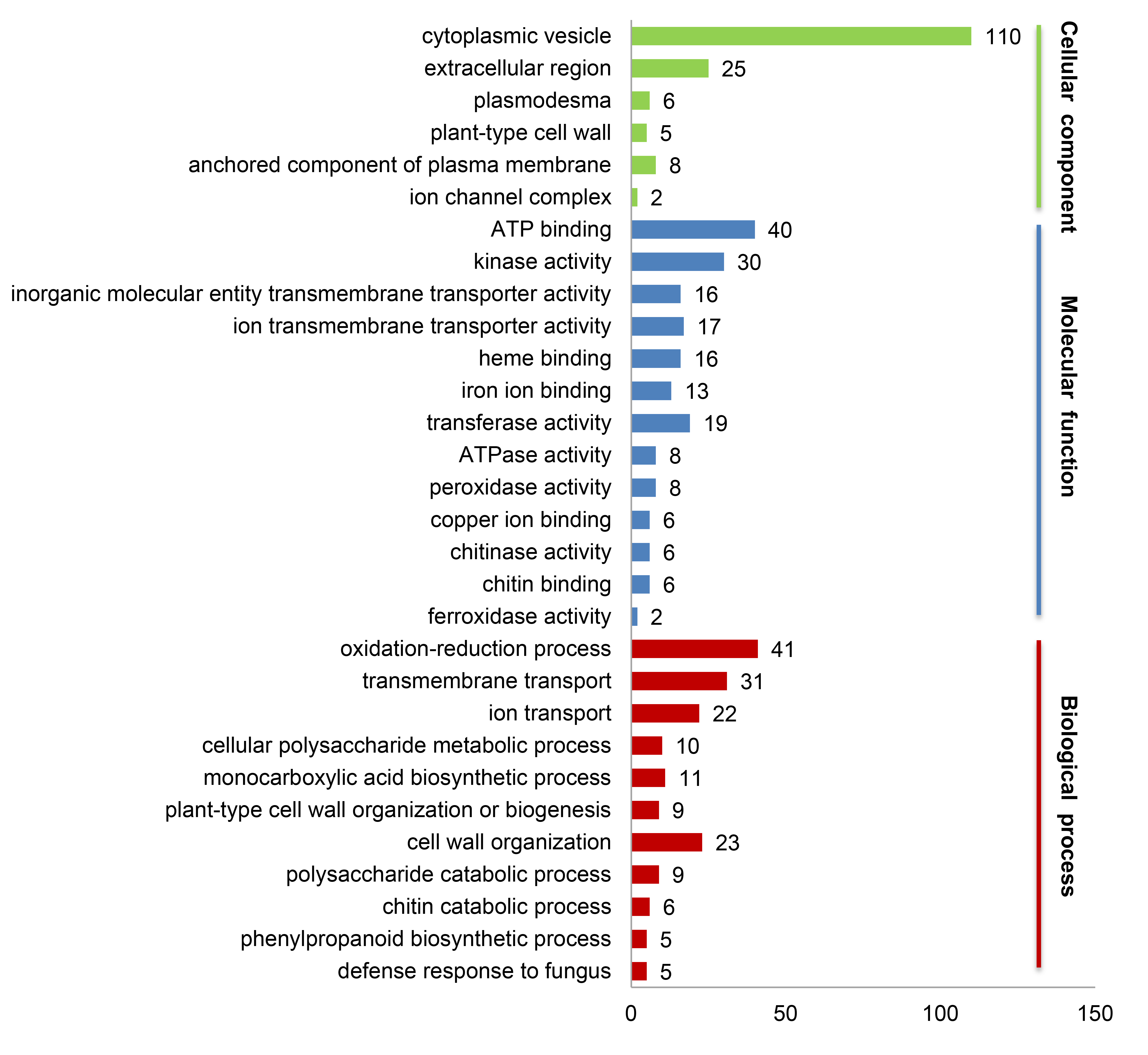

Supplement: Supplementary file 14 — Additional file 14: Figure S8. GO analysis of DEGs uniquely regulated in the OsPGIP1-overexpressing rice. The GO analysis of DEGs that specifically changed in OsPGIP1 OV-24-RS included cellular component, molecular function and biological process categories. [file 12284_2019_352_MOESM14_ESM.jpg]

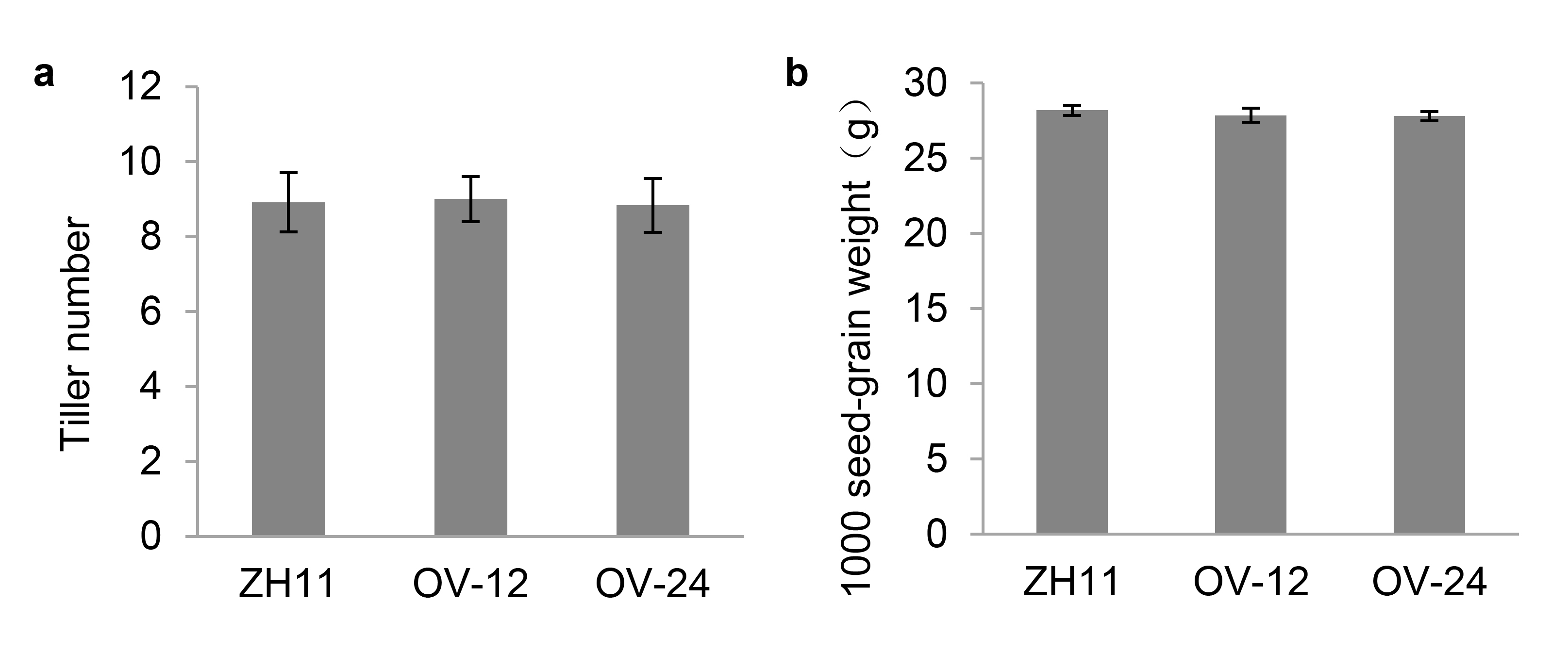

Supplement: Supplementary file 15 — Additional file 15: Figure S9. The yield traits of OsPGIP1-overexpressing rice showed no significant changes. (a) The tiller number of the two OsPGIP1 OV lines (OV-12 and OV-24) and ZH11 were counted in at least 30 individual plants after the full growth period. (b) The OV-12, OV-24 and ZH11 rice seeds were harvested after the complete growth period and after removing moisture with a dryer. Then 1000 seed grains were weighed for the rice, and the experiment was repeated 10 times. [file 12284_2019_352_MOESM15_ESM.jpg]
